# Transmission of an environmental modification in quails across three generations: changes in the contribution to phenotypic variability

**DOI:** 10.1186/s12711-026-01079-0

**Published:** 2026-08-10

**Authors:** Stacy Rousse, Sophie Leroux, David Gourichon, Ludovic Calandreau, Andrea Rau, Sandrine Lagarrigue, Tatiana Zerjal, Frédérique Pitel, Sonia E. Eynard

**Affiliations:** 1 GenPhySE, Université de Toulouse, INRAE, ENVT, 31326 Castanet-Tolosan, France; 2 PEAT, UE1295 - INRAE Centre Val de Loire, 37380 Nouzilly, France; 3 Centre Val de Loire, UMR Physiologie de La Reproduction Et Des Comportements, INRAE, CNRS, IFCE, Université de Tours, 37380 Nouzilly, France; 4 GABI, Université Paris Saclay, INRAE, AgroParisTech, 78350 Jouy-en-Josas, France; 5 PEGASE, INRAE, Institut Agro, 35590 Saint Gilles, France

## Abstract

**Background:**

While epigenetic variations can contribute to shaping phenotypic diversity, it can be challenging to isolate and quantify the portion of trait variability under non-genetic influence. In this study we compared the phenotypic responses for different traits of two epilines of Japanese quails (*Coturnix japonica*) across three generations, using a large sample size. These epilines were built in parallel following (epi +), or not (epi -), an initial genistein ingestion in the ancestors' diet and were maintained to harbour a similar genetic structure.

**Results:**

Linear models were fitted to extract the fraction of variance allocated to multiple factors such as family, sex and epiline. The latter was found to be significantly associated with body weight. The contribution of the epiline to phenotypic variability progressively increased from the first generation (G0) to the last (G2), leading—for example in body weight at slaughter—to an average difference for adult males and females in G2 of 9 g and 14 g respectively, between epi + offspring and controls (epi-).

**Conclusions:**

Although these findings suggest genetic drift, they could also reveal a possible transgenerational effect of the initial diet disruption. The analysis of other phenotypes displayed rare significant effects of the epiline. This innovative experimental design offered a unique opportunity to better understand the evolution of phenotypic variability and the parameters constituting it across three generations following or not an environmental change.

**Supplementary Information:**

The online version contains supplementary material available at 10.1186/s12711-026-01079-0.

## Background

The increased frequency of environmental constraints due to climate change (e.g. heat waves, resource limitation …) can be a source of stress for animals. One of the key drivers of adaptation is the emergence of new phenotypes, which arise from the combined effects of genetic influence of parental alleles and non-genetic factors. Fluctuations in the parental environment can trigger non-genetic modifications, such as microbiota, learning or epigenetics, resulting in changes in gene regulatory mechanisms and thus gene expression, even in the progeny [1, 2]. Understanding the impact and transmission of environmental effects to subsequent generations is crucial to comprehend how animals adapt to changing environments to maintain animal production needs while ensuring animal welfare. The study of a change in offspring phenotype, caused by an environmental signal experienced by the parental generation (and possibly previous ancestors), without involving genetic change in the offspring can be referred to as transgenerational plasticity in certain research fields [3].

Most studies classically tackle the direct impact of the environment on the traits of exposed animals and on the performances of the subsequent generation. However, others have managed to evaluate a possible transgenerational non-genetic inheritance, considering an environmental effect to be transgenerationally transmitted when the individuals exhibiting trait variations were not exposed to the environmental stressor, even as germ cells [4]. On the contrary, an effect is considered as intergenerationally transmitted when trait variations are observed in the offspring of the individuals exposed to an environmental modification, therefore in an individual exposed as embryo or germ cell status. Multigenerational, including inter- and transgenerational, studies in farm animals are challenging due to the need to raise multiple generations. To this end, Japanese quail (*Coturnix japonica*) is an ideal avian model for multigenerational analysis, thanks to its short generation interval, the wide range of measurable phenotypes, and the controlled breeding practices and limited space in which it can be reared. Previous studies on a variety of traits such as body weight, egg production and behavioural characteristics using Japanese quails as model organisms have reported multi and transgenerational phenotypic responses of the progeny after a thermal increase in the parental embryonic environment [5], after in-ovo injection of genistein, an endocrine disruptor [6], and after exposure to maternal stress [7]. These studies suggest a possible transmission of environmental effects through changes occurring in the prenatal embryonic environment. In addition, some evidence of the importance of grandmother’s diet was documented in mule and Muscovy ducks, with significant variations in ducklings and adult weights after methionine deprivation in the diets of the grandmothers [8]. This suggests that diet disruptions can indeed be a cause of phenotypic response to an unknown yet memorised environment, through a specific inheritance mechanism.

In light of these previous analyses, we aimed to expand upon the pilot study and deepen our understanding of potential multigenerational effects. In the initial study [6], the authors established two quail populations originating from a common founder line and differing only by an embryonic genistein exposure, using a mirrored single-pair mating design to minimize between-line genetic divergence. Genistein is a phytoestrogen compound naturally present in soy, and was notably associated with significant shifts in DNA methylation profiles in the promoter region of the Agouti gene in mice [9]. Among non-genetic factors influenced by the environment, DNA methylation, an epigenetic phenomenon, can be involved in the construction of phenotypes [10]. Several studies in mice and humans have demonstrated the influence of genistein on DNA methylation, including in embryonic stem cells (e.g. [11–13]). Genistein is also known, among others isoflavones, to affect performances in livestock, including poultry (see [14]).

As genistein has been shown to induce stable DNA methylation changes and to affect several traits in different species, it was considered as a relevant chemical compound to study environmentally induced epigenetic variation in a livestock species. The pilot experiment showed that the embryonic environment can affect growth, reproductive and behavioural phenotypes three generations later and was associated with changes in blood DNA methylation [15], thereby suggesting a contribution of non-genetic inheritance mechanisms. The two populations, issued from treated or control ancestors, were named 'epilines' to emphasise the potential epigenetic inheritance of environmental influences during development, even if such putative non-genetic transmission may be due to other non-genetic processes such as cytoplasmic, microbiotal or behavioral transmission [1, 16, 17]. However, in this pilot experiment, genistein was directly injected into the egg and intermediate generations were not phenotyped or characterised at the molecular level, which limits inference to realistic field-like conditions and prevents a fine dissection of multigenerational responses.

In addition, the use of genistein as supplementation within the feed of Japanese quails has been shown to lead to better adaptation of birds to heat stress by stimulating feed efficiency and growth rate [18]. We therefore designed a multigenerational quail experiment in which maternal dietary genistein supplementation acts as the environmental exposure. This design aims to more closely mimic living conditions (ingestion rather than injection), while generating populations suitable for joint genetic and non-genetic analyses across all generations up to F3. We hypothesised that maternal genistein supplementation would induce specific phenotypic and epigenetic changes in directly exposed offspring, and that part of these changes would persist in unexposed descendants, consistent with non-genetic inheritance of environmentally induced variation.

While epigenetic variations can contribute to shaping phenotypic diversity in offspring, isolating and quantifying the portion of trait variability influenced by epigenetics remains challenging. To date, although genetic contribution has largely been documented for a wide range of traits in a large number of poultry species, no direct quantification of the epigenetic influence on these traits has been reported. The environment usually accounts for a large part of the observed variability depending on the phenotypes measured, which translates to low heritabilities even under selection [19–24] providing a compelling reason to estimate the contribution of non-genetic factors, including epigenetics, to trait variability. In this study, a case-control design allowed building two distinct epilines. The epiline information provides an indirect way to estimate the epigenetic contribution to the trait, as well as other non-genetic factors. By investigating the multigenerational transmission of the effects of an initial diet disruption across three generations of quail, we aimed to capture the inter- and transgenerational impacts of environmental influences, likely mediated by non-genetic mechanisms. The portion of phenotypic variability linked to an ancestor’s environmental change and its evolution across generations was estimated in body weights.

## Methods

### Quail mating design

**Fig. 1:**
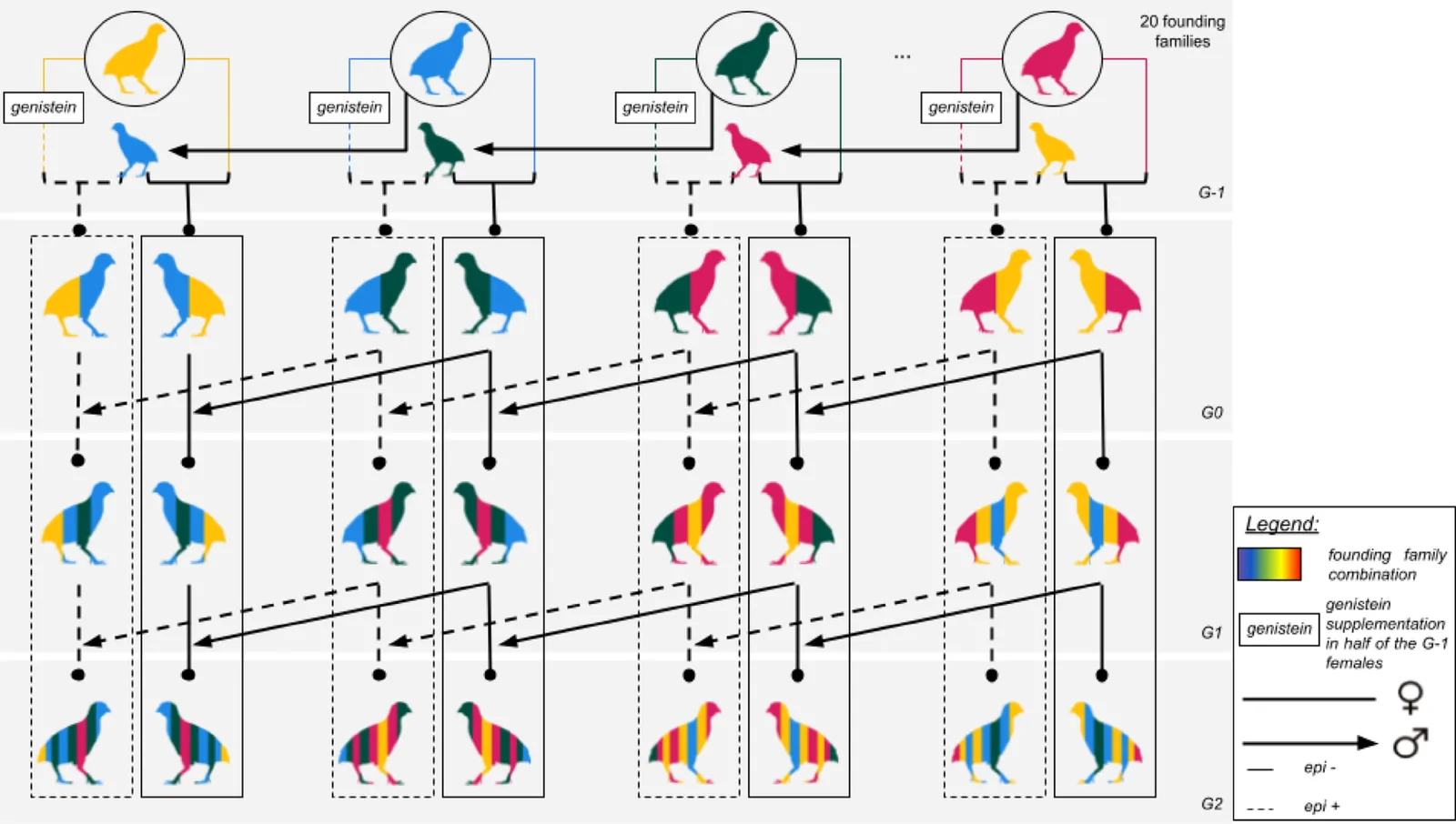
Quail mirror mating design. The quail mating design runs over four generations of quails, the first one (G-1) containing the founding populations that have been split into two populations from G0 onwards and mated independently for three successive generations. Genistein capsules were administered to females in G-1 while their full-sib female counterparts were provided empty capsules. Both dams were then mated with the same male coming from another genetic background. The G0 progeny was divided in two epilines (epi + deriving from the dams having ingested the genistein and epi- deriving from the control dams) of similar genetic background. The following matings were controlled to respect the balance of the genetic contribution from founders by ensuring a mirror between epilines (solid lines: epi- and dashed lines: epi +). The theoretical genetic contribution of founders is represented by the colors

All animals in the experiment come from a Japanese quail (*Coturnix japonica*) experimental line (HSR, for High Social Reinstatement) arising from a divergent selection on social motivation [25]. From this common genetic background, twenty initial founding families were crossed, producing the generation G-1. In each founding family, two full-sib females were mated at maturity with the same male, originating from another family. One of the females was administered a genistein capsule (100 mg, Molekula group) for thirty days after mating while the other one was only provided with empty capsules, leading to the development of G0 individuals in two different embryonic environments. The females that received the genistein are the dams of the treated epiline (thereafter called epi +), in opposition to the control epiline (epi-). After this initial diet disruption, the quails were mated following a mirror design, with males and females from the same founding families mated in each line, to maximize the genetic balance between the two epilines. The goal of this mating design is to maintain the quails in both groups as genetically similar as possible while ensuring that the only potential difference between them is the initial dietary disruption. Therefore, if phenotypic variability is observed between the two groups, it can be hypothesized that it results from the change in feed intake during the first generation and limits the possibility that it arises from a genetic factor. Groups were then maintained under standard rearing conditions, at the breeding facility, for the three subsequent generations (G0, G1 and G2), with water and feed ad libitum (Fig. 1). The ‘epiline’ terminology is therefore used to refer to these two parallel groups of quails whose distinction lies within their non-genetic factors, that includes epigenetic mechanisms per se as well as any other environmental effects that can be transmitted across generations. From hatching to 49 days-of-age, individuals were floor-raised on litter altogether, in climate-controlled housing. Animals were then placed within cages of two individuals of same sex, until the age of twenty weeks, when they were coupled for mating. All diet, temperature and light conditions were standard and the same for all animals, all reared in the same barn.

### Phenotypic traits

Phenotypic records were collected over four generations for body weights, tissue weights, production and behavioural response to isolation.

#### Body weights (BW)

Body weights were measured at different developmental stages: one week, four weeks, seven weeks and slaughter (thirty weeks of age on average) for all quails. A unique scale was used for all measurements and weights were collected at the same age when possible to ensure consistency between generations. Slaughter was done through electronarcosis immediately following the recording of the final body weight measure.

#### Tissue weights (TW)

Several tissues were weighed: abdominal fat (for all generations), heart, liver (for G0 onwards), and testicle (for G1 onwards). All weights were adjusted by correcting for the weight of the quail at slaughter.

#### Egg production (PROD)

At twenty weeks of age, quails were placed in cages for reproduction. Egg production data, total egg number during the laying period and precocity, were recorded from the first day in the cage for 49 days. The laying rate was estimated as the number of eggs laid over the laying period. The mean egg weight per quail was calculated on a basis of four eggs collected and weighed per quail.

#### Behaviour at isolation (BHV)

Adult quails underwent an open field behavioural test in which their reactivity to a new environment was assessed. Each quail was placed in a cubic arena (80 cm) of white wood with a floor made of beige linoleum, in an unknown experimental room surrounded by an unknown white curtain. Each quail was individually placed at the centre of the arena and allowed to freely explore it for 300 s. For each trial, the time spent in the inner area (a square of 20 cm × 20 cm at the centre of the arena), the frequency of time spent at the periphery (outside the inner area) as well as the total distance covered by the quail was recorded by a camera placed above the arena. The open field test was performed for all generations.

### Statistical analysis strategy

The phenotypes were analysed following these steps: first, we performed a descriptive overview of the phenotypes by looking at the overall trait distribution for all raw phenotypes, the distributions per generation and sex within each epiline are shown in Additional file 1 Figures S1-S2; second we tested normality; third we looked at the potential impact of the epiline factor by mean comparison; fourth, we applied some transformations on our phenotypes of interest when necessary; and at last we ran a linear model to obtain an estimation of the part of variability explained by different factors of interest.

#### Normality and mean comparison tests

Shapiro–Wilk normality tests were performed on all raw phenotypes (Additional file 2 Table S1). Since all variables did not meet the normality assumptions, Kruskal–Wallis (KW) tests were applied to perform pairwise mean comparisons between epilines. Given the strong sexual dimorphism in quails, the KW test was applied to all raw phenotypes in each sex and generation separately, therefore leading to a total of 71 tests. False Discovery Rate (FDR) correction was applied to account for the multiplicity of tests. The null hypothesis H0 “No significant effect of the epiline on the phenotype” was rejected at the 5% level of significance. The results are shown in Additional file 2 Table S2.

#### Data normalization

To avoid biased estimates of coefficients and meet the assumptions of linear modeling, we calculated the skewness of all traits following different transformations using the R ‘moments’ package (version 0.14.1) [26]. Skewness of Log10, square root and inverse transformed data were compared to that of the raw data and the transformation associated with the lowest skewness value was subsequently retained for each trait (Additional file 2 Table S3). The choice of transformations tested were selected among commonly applied data transformations prior to linear modeling [27].

#### Estimation of the contribution of parameters to body weight traits

Following the results of Kruskal–Wallis tests, we decided to focus our analyses on body weight traits (results on other traits are shown in Additional file 3 Tables S4-S5 and Additional file 4 Figures S3-S4). To estimate the percentage of variability explained by influential factors such as the family, sex and epiline, linear models were fitted to normalized body weights.

#### Linear models (LM)

For each body weight at each generation, the optimal set of parameters was first determined with a stepwise model selection using the stepAIC function of the R ‘MASS’ package (version 7.3–65) [28]. A linear model was fitted for each trait as follows:

$$ y = 1\mu \, + X\beta + \varepsilon , $$

where **y** is the phenotype, **µ** is the overall mean, **X** is the design matrix for the fixed effects, **β** is the vector of fixed effects and **ε** is the residual. Fixed effects included in the model were sex (male, female), epiline (epi-, epi +) and founding family combination (see Fig. 1) and their pairwise interactions (sex:epiline, sex:family and epiline:family). Effects were iteratively added or deleted until the model with the lowest Akaike information criterion (AIC) was identified for each trait. The LM fit on all traits is shown in Additional file 3 Table S4.

#### Cross validations

After selecting the optimal model, we evaluated the fit of the model using 100 random fold cross validations (80% of data in the Train set, 20% in the Test set). For each fold, the Train set was used to fit the model using the optimal equation determined with stepAIC, and it was then used to predict the phenotypic response of the Test set. Correlations between observed and predicted Test phenotypes were then assessed. The coefficient of determination R^2^, which represents the overall variance of the trait captured by the model, was computed for each fold.

#### Effect of parameters

The proportion of phenotypic variability explained by each factor for a given trait was estimated using an analysis of variance (ANOVA – type III). The total sum of squares SS_Total_ is decomposed into the sum of squares for each factor j SS_Factor_ and residual variance SS_res_. The proportion of variance explained (PVE) of factor j may be calculated as:

$$PVE_{j} = \frac{{SS_{Factor_j} }}{{SS_{Total} }}, $$

Estimates were obtained using the R package ‘FactoMineR’ (version 2.12) [29] with Type III sum of squares. We ensured success in variance components estimations by retaining only families with a sufficient number of quails per epiline.

All statistical analyses were performed using R 4.5.2 (R core team) [30].

## Results

The summary statistics of all traits collected from all generations are available in Additional file 5 Table S6.

### Impact of the epiline on phenotypes and evolution across generations

#### Body weights

**Fig. 2:**
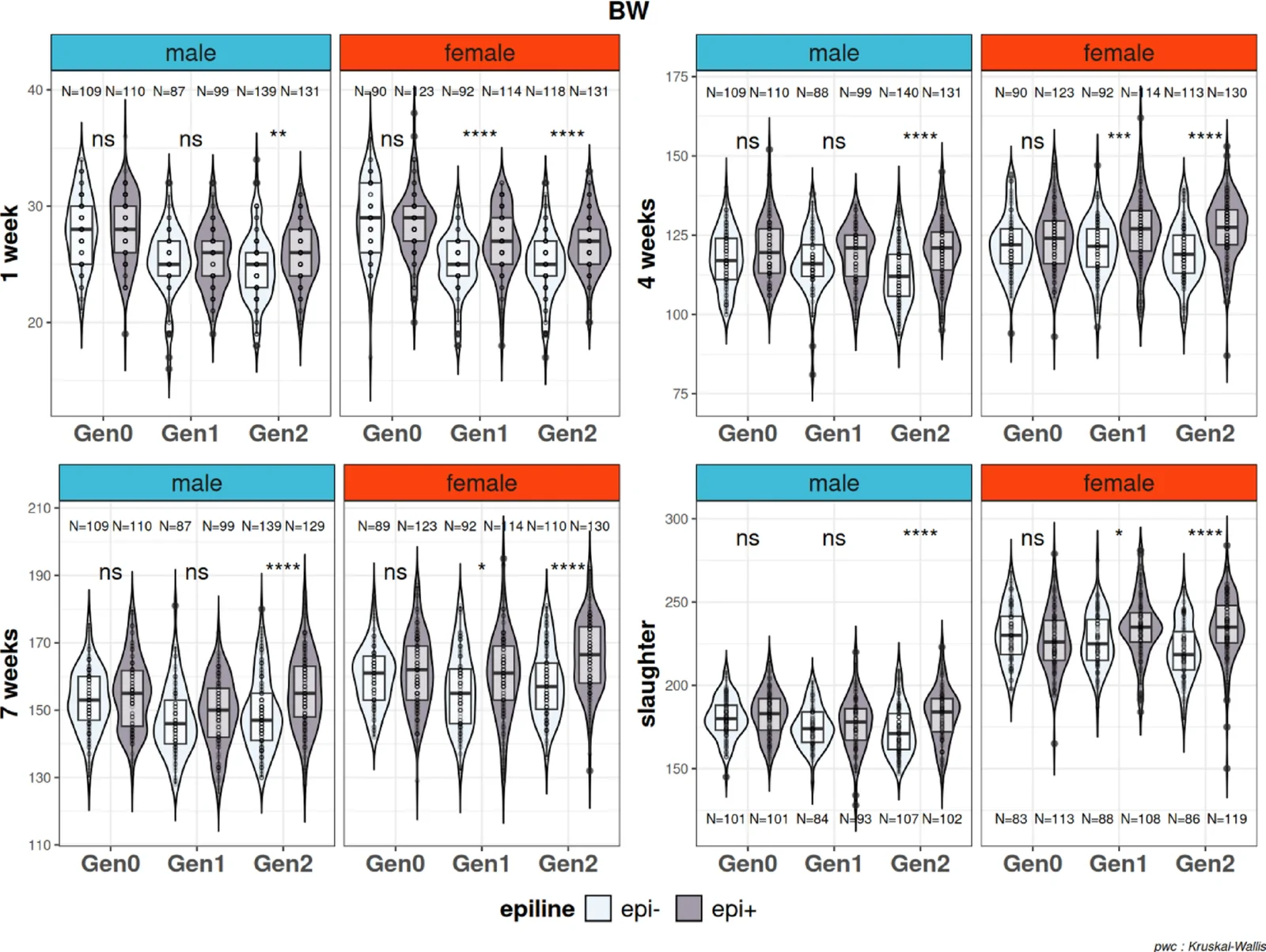
Mean comparison of body weights at one week, four weeks, seven weeks and at slaughter (thirty weeks) between epilines. Kruskal–Wallis tests were performed to identify differences between epilines for each phenotype, on raw estimates, for each sex and generation independently. N values indicate sample sizes within each sex and epiline per generation. The asterisk indicates the level of significance of the Kruskal–Wallis test results after FDR correction for multiple testing, ns *p*-value > 0.05, * *p*-value < 0.05, ** *p*-value < 0.01, *** *p*-value < 0.001 and **** *p*-value < 1e-4

Significant differences between epilines were observed across several body weights. Females showed significant differences starting from generation G1 at all ages with epi + quails systematically weighing more on average than the epi-controls. The same pattern was found in males where the groups began to consistently diverge in G2 at all ages; however, no significant difference was observed in earlier generations (Fig. 2).

Differences in average BW at all ages became increasingly more pronounced over the generations. In G0, the difference between epi + and epi- at slaughter was on average 4 g for both sexes, which was not found to be significant. In G1, the difference between epilines for females was on average 7 g and reached 14 g in G2. In males, a similar pattern was observed in G2, with epi + quails weighing 9 g more on average at slaughter whereas a maximum difference of 2 g was observed in earlier generations. The growth curve to highlight this longitudinal evolution is added in Additional file 6 Figure S5.

#### Other phenotypes

For other phenotypes, such as TW, PROD or BHV, significant differences between epilines were infrequent and sometimes linked to individual singularities (adjusted heart weight in G2 males, precocity in G1 females). Adjusted heart weight in G1 and G2 males as well as precocity in G1 females were the only traits for which a significant difference between epilines was detected. These results are displayed in Additional file 7 Figures S6-S8.

### Body weight variability explained by parameters: evolution across generations

#### Effect of the epiline

**Fig. 3:**
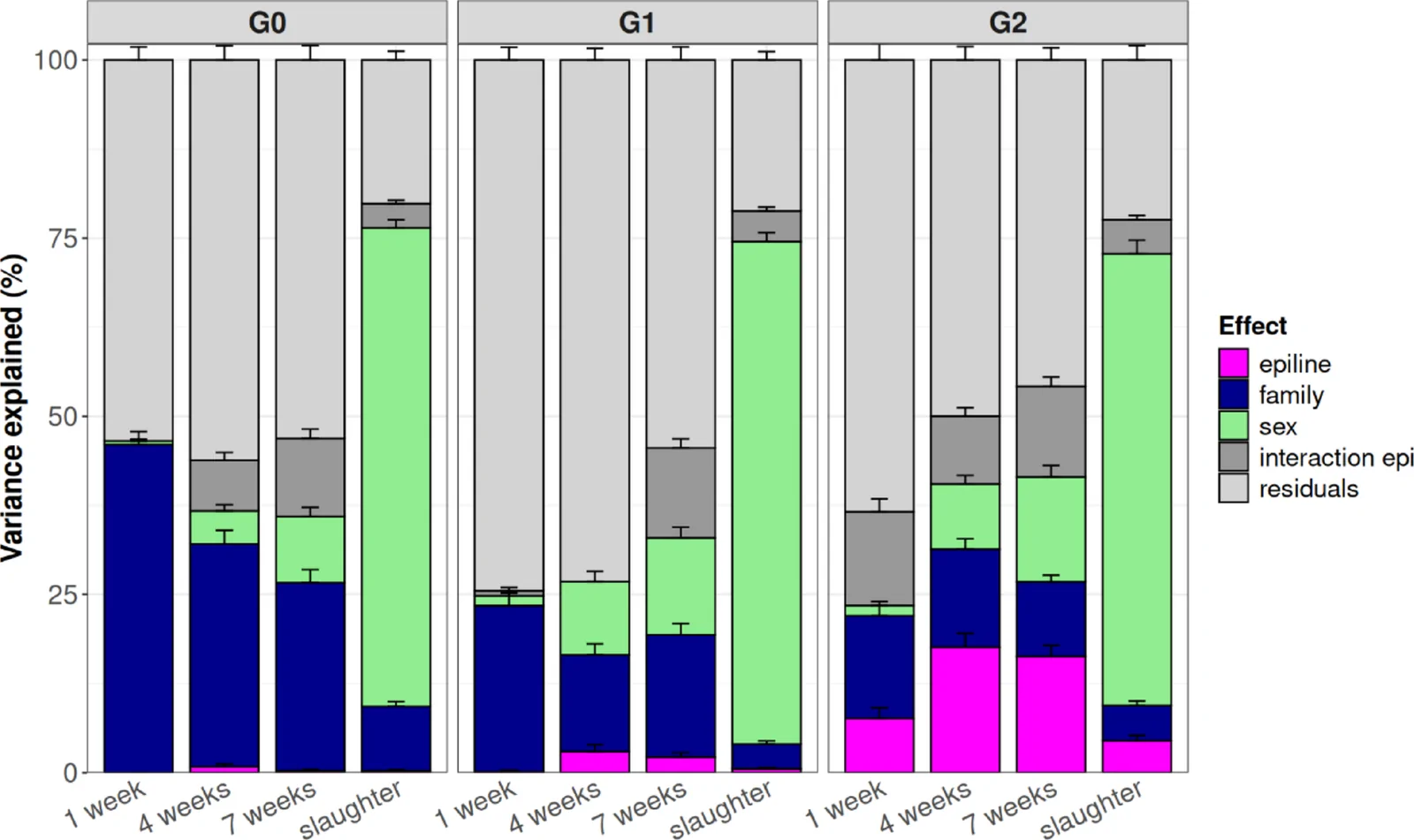
Contribution of parameters (sex, family, epiline, and interaction) to body weight variability (in percentage) per generation. Proportion of phenotypic variability explained by the effect of sex, family, epiline and their interaction as extracted from the best linear model for body weight traits. The interaction parameter always involved the epiline (hence the terminology ‘interaction epi’ used). More specifically, it refers to: epiline:family for BW at 1 week (G2), BW at 4 weeks (G0, G2), BW at 7 weeks (G0, G1, G2) and BW at slaughter (G1, G2); sex:epiline for BW at 1 week (G1); sex:epiline and epiline:family for BW at slaughter (G0). Body weights throughout growth are arranged within generations G0, G1 and G2

Model fitting revealed that the epiline explained a different portion of the variability observed in BW according to the generation and age of the quails. The epiline effect was found significant from four-weeks old quails onwards, in all generations. First, at one week of age, results showed significance in G2 only, with the epiline explaining 7.6% of the variability. Then, for four- and seven-weeks old animals, the epiline explained a negligible amount of weight variability in G0 (under 1%), but it was found to increase in later generations, accounting for around 2% in G1, to finally reach a more substantial proportion in G2 (over 15%) at those ages. Finally, the contribution of the epiline to BW in adults (BW at slaughter) was much lower than at previous ages (less than 1% in G0 and G1 and 4.5% in G2) (Table 1, Fig. 3).

**Table 1:** Estimates of the contribution of parameters (and standard deviation of replicates) to body weight variability (in percentage)

Trait	Generation	epiline	family	sex	interaction	residual
1 week	G0	0 (0)	46.0 (1.8)	0.5 (0.3)	0 (0)	53.4 (1.8)
	G1	0.1 (0.2)	23.3 (1.8)	1.4 (0.5)	0.7 (0.4)	74.4 (1.8)
	G2	7.6 (1.5)	14.4 (1.4)	1.4 (0.5)	13.2 (1.8)	63.4 (2.3)
4 weeks	G0	0.8 (0.4)	31.3 (1.9)	4.6 (0.9)	7.1 (1.1)	56.2 (2.0)
	G1	3.0 (0.9)	13.5 (1.6)	10.3 (1.4)	0 (0)	73.2 (1.7)
	G2	17.6 (1.9)	13.8 (1.4)	9.1 (1.2)	9.5 (1.1)	50.0 (1.9)
7 weeks	G0	0.3 (0.2)	26.4 (1.9)	9.3 (1.3)	10.9 (1.3)	53.1 (2.0)
	G1	2.2 (0.6)	17.2 (1.6)	13.6 (1.5)	12.6 (1.3)	54.4 (1.8)
	G2	16.3 (1.6)	10.5 (0.9)	14.7 (1.6)	12.7 (1.3)	45.8 (1.7)
slaughter	G0	0.3 (0.1)	9.0 (0.7)	67.1 (1.1)	3.4 (0.5)	20.1 (1.2)
	G1	0.5 (0.2)	3.5 (0.4)	70.5 (1.2)	4.3 (0.5)	21.2 (1.1)
	G2	4.5 (0.8)	4.9 (0.6)	63.4 (1.9)	4.8 (0.6)	22.4 (2.0)

Epiline, family, sex, interaction and residual components of each trait are expressed in percentage with the standard deviation in brackets. The estimates were calculated from the extraction of the sum of squares from analyses of variance (ANOVA type III) results. All figures are percentages and represent the average contribution of the different factors to phenotypic variability using the estimates obtained across the 100 folds of cross validations. The interaction parameter refers to: epiline:family for BW at 1 week (G2), BW at 4 weeks (G0, G2), BW at 7 weeks (G0, G1, G2) and BW at slaughter (G1, G2); sex:epiline for BW at 1 week (G1); sex:epiline and epiline:family for BW at slaughter (G0)

#### Effect of other factors

Contrary to the increase of the epiline effect across generations, the portion of phenotypic variance explained by the family declined, starting, at one week of age, from 46% in G0 to 23% in G1 and decreasing again to 14% in G2 (Table 1).

The family effect was also stronger in younger animals. It ranged from 46 to 9%, from 23% to 3.5%, and from 14.4% to 5% between one week of age and slaughter for G0, G1 and G2 respectively. On the other hand, the proportion of variability explained by the sex always increased with animal age. It ranged from 0.5% to 67%, from 1.4% to 70.5% and from 1.4% to 63.4% between one week of age and slaughter for G0, G1 and G2 respectively. The interaction parameter, when conserved, always involved the epiline and the family, except in two traits: in body weight at one week in G1 where it concerned epiline and sex, and in body weight at slaughter in G0 where it concerned epiline and sex as well as epiline and family. Depending on the generation and the trait, this parameter showed little to moderate contribution to phenotypic variability, ranging from 0 to 12%. Overall, the residual remained high in all traits but BW at slaughter, regardless of the generation.

#### Cross validations

**Fig. 4:**
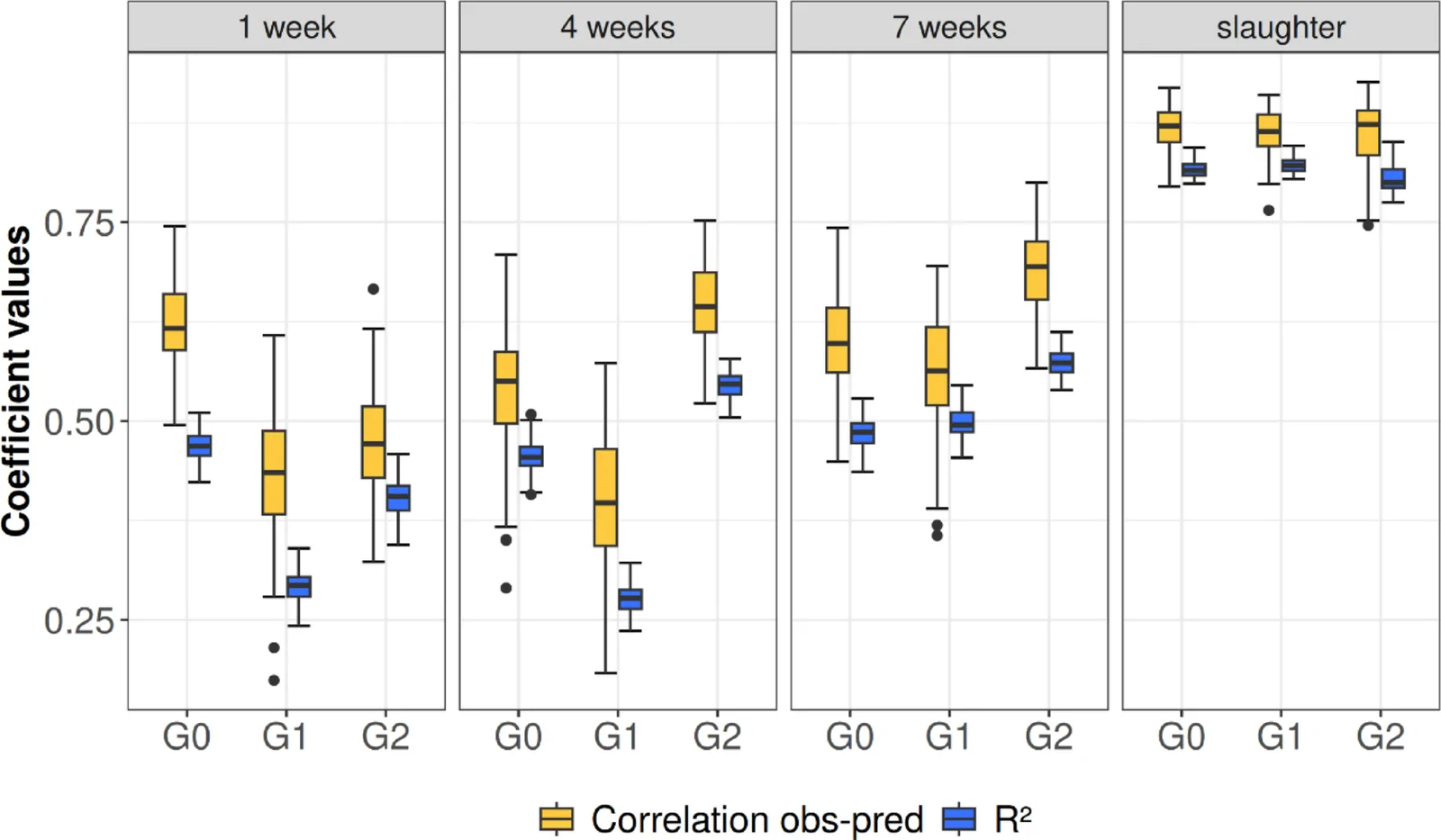
Model robustness (R^2^) and correlations after cross validations. The coefficient of determination R^2^ (blue) and the correlation between observed and predicted values in the Test set (yellow) were determined in 100 cross validations, to evaluate the predictive performance of linear models. The results show the distribution of R^2^ and correlation values in each of the 100 cross validations, and are displayed per generation (x-axis) for each body weight (1 week, 4 weeks, 7 weeks and at slaughter)

The predictive performance of the models, evaluated using cross validations, varied considerably across BW traits. Body weight at slaughter consistently exhibited the highest prediction accuracy across all generations (R^2^ ≥ 0.8, Corr ≥ 0.8), highlighting good model performance for adult individuals. For younger animals, the coefficients of determination were significantly lower than at slaughter despite remaining of reasonable magnitude (R^2^ ≤ 0.5 in G0 and G1 and R^2^ ≤ 0.6 in G2). The correlations between observed and predicted values for the Test sets were moderate, ranging between 0.4 and 0.6, suggesting limited predictive power of our models for these traits; these results are in line with the large residual variance estimated (Fig. 4).

#### Other phenotypes

Results on the estimation of the effects of parameters on the variability of other traits are shown in Additional file 4 Figure S3. The epiline was a significant factor to keep for explaining a portion of phenotypic variability for several traits. In adjusted tissue weights like heart, abdominal fat and testicle, this parameter was kept after the stepAIC in all generations. For the liver, it was only found significant in G2. For PROD traits, the epiline was found significant in precocity and in adjusted egg weight at every generation but not for the laying rate. Finally, in behavioural traits, the epiline factor was kept in the models for total distance and frequency at periphery in G1 and G2 only, whereas it was kept in G0 and G1 for duration at centre. Although the epiline appears as a significant factor in the aforementioned traits, the estimates of the portion of variability explained remain below 5% in all, and negligible in most (inferior to 1% in adjusted abdominal fat, adjusted liver weight, total distance, frequency at periphery and duration at centre). Besides, for all the traits presented in Additional file 4 Figure S4, a substantial part of phenotypic variance remains uncaptured by our model, highlighting a strong limit in the explanatory power of our models and a limited confidence towards the estimates of the portion of phenotypic variability explained by the epiline for these traits.

## Discussion

The aim of this study was to analyse phenotypic variations across three generations of Japanese quails (*Coturnix japonica*) following supplementation with genistein, an endocrine disruptor. For this purpose we built one of the largest available designs in livestock containing groups impacted (treatment) or not (control) by environmental stressors and multiple generations of mirror breeding to limit the impact of genetic variation in the determination of trait changes through time.

### Genistein ingestion is associated with different phenotypic responses across generations

In both sexes, BW tended to increase in the treated epiline (epi +) compared to the control epiline (epi-), with the difference between epilines becoming progressively more pronounced from generation G0 to G2. In a pilot study [6], a similar pattern was observed in G2 females: genistein injection into the egg was associated with increased BW at three weeks of age. However, in that study, the effect was reversed in males (genistein injection associated with decreased BW). In the present study, we did not observe such sex-specific interactions regardless of the generation or age considered. On the other hand, the results obtained here concur with other studies regarding the significant impact of genistein on BW. The scientific community remains divided on the impact of the phytoestrogen on the organism [31, 32], with most studies tackling the direct effect of ingestion on the concerned individuals. More generally, body weight management is of importance in poultry, as this trait often correlates with production and reproductive efficiency [33]. The choice of feed can be critical to optimize carcass related traits in broilers' offspring [34] as well as ensuring ideal reproductive performances [35]. In that regard, our results on body weight divergence provide insight on the need to vigilantly monitor feed intake as well as feed source, according to the breeding strategies at stake.

PROD traits did not exhibit the strong epiline-related effects seen previously in the pilot study, where epi + females began laying eggs more than eight days later than control females on average. Likewise, abdominal fat weight and BHV traits did not demonstrate the previously reported significant differences between epilines. The discrepancies between the two studies, despite using the same quail genetic line and the same endocrine disruptor, are likely attributable to differences in the method of genistein administration: in the pilot study, 500 µg of genistein were directly injected into the egg before incubation, whereas in the present study genistein was administered orally to the G-1 mother to mimic more realistic breeding conditions, resulting in substantially lower concentrations of the molecule in the egg [36].

Direct effects of genistein ingestion on phenotypes have been reported in humans and animal models. Although this molecule offers promising results in cancer treatment [37, 38], soy overconsumption has demonstrated harmful effects on reproductive [39–42] or cognitive [43] functions. These effects of endocrine disruptors on health seem to be persistent over several generations, although this remains relatively unexplored, despite a few specific studies [44]. The molecular effects of this phytoestrogen have been characterized, especially in cancer research, genistein acting similarly to estrogens, but also as an antioxidant, anti-inflammatory agent and inhibitor of cell cycle regulators (see [45, 46] for a review). These studies also underline the low bioavailability of the molecule, especially in its glycosylated form, as reported for other flavonoids [47]. A similar issue arises in quail, where it was observed that glycosylation reduces the transfer and accumulation of genistein in egg yolks after maternal supplementation [36], indicating that the chemical form and dose influence embryonic exposure. Despite these pharmacokinetic constraints, it has long been known that supplementing the diet of broilers with soy isoflavones, including genistein, improves growth performance, especially at low doses (< 80 mg/kg) [48, 49].

Maternal dietary supplementation with genistein has also been shown to affect offspring growth: in broilers, low maternal doses (e.g. 40 mg/kg) increase embryo weight, while higher doses (e.g. 400 mg/kg) can decrease it, indicating a non‑linear, dose‑dependent response [50]. In quail, our previous work using direct egg injection of genistein, which corresponds to a high dose in the embryonic environment (500 µg), showed reduced body mass at several ages, confirming that high developmental exposure can alter growth trajectories [51]. The divergence in body weight trend observed between the pilot study [6] and our study confirm the dose effect on this trait. In fact, the injection practiced in [6] led to a reduction in offspring weight for the treated group whereas the ingestion, in this study, shows an increase. Adding genistein to the maternal diet has been shown to increase genistein concentration in egg yolks, in Japanese quail, confirming that the molecule may reach the embryo through the yolk [36, 52]. Our oral maternal route thus ensures realistic embryonic transfer via yolk, supporting phenotypic effects while allowing tests of non-genetic inheritance—our core question.

Overall, results reveal different sensitivities of the phenotypes towards genistein ingestion. Although this molecule is already used as a supplement to increase BW in broilers [50], it has been observed that it affects other phenotypes as well, but not as strongly [53].

### Missing variability highlights the complexity of phenotypes

The proportion of phenotypic variance explained by the family displayed a clear trend with age, decreasing over the lifetime (with the exception of week 7 in G1). In our model, we accounted for a family factor (founding family combination described in Fig. 1) as a way to include the current and ancestral genetic effect and genetic composition in each of the two epilines. This suggests that the influence of the inherited genetic background becomes progressively less determinant as individuals age. Conversely, the contribution of the sex factor increased with age, consistent with the emergence of pronounced sexual dimorphism in quail. These patterns illustrate that different factors influence animal phenotypes in distinct ways at different stages of life, and that the proportion of phenotypic variance explained by a given factor is therefore strongly dependent on the individual’s age at the time of measurement.

Nevertheless, residual variance was substantial across all body weights and this is even more valid for other traits. This is highlighted by the generally moderate values of coefficient of determination (R^2^), suggesting the difficulty of the models to accurately reflect the overall variability of BW, especially in early life. The R^2^ values for body weight at one week (< 0.5 in all generations) suggest limited explanatory power of the model. Given the low effects of the epiline estimated for this trait, the reliability of the estimation is questionable. In poultry, a major effect of the dam is expected to influence most early-life traits of the offspring through egg size and composition [54, 55]. In our model, this dam effect is absorbed within the family factor, and our results reveal that it is the factor which contributes most to body weight variability at one week of age, for all generations. So, in spite of its limited explanatory power, the model seems to show coherent trends with biological meaning. Over time, especially after four weeks of age, R^2^ increases slightly showing a better suitability of our model. A potential explanation for the large residual variance relies on the complex determinism of phenotypic traits collected, influenced by various environmental factors, some of which the design may have failed to eliminate despite the controlled management. Multiple studies also reported high residual variances remaining, for example on a New Zealand bird species, a study found that home-range sharing and genetic relatedness among individuals contributed to phenotypic similarities for several morphological traits, yet residual variance remained high [56]. Similarly, Béziers et al. [57] reported high residual variance for stress-induced corticosterone levels in a barn owl population, hypothesizing an additional factor beyond genetics or nest environment: the experienced environment. Since all individuals from the same generation in our study were reared under identical conditions, a plausible explanation for part of this residual variance would be the transmission of the experienced environment from the first generation, possibly involving slight variations in genistein dosage among eggs. This would come in addition to the weight of the expected random variations on the residual variance. The embryonic environment is known to influence the individual phenotype later in life, especially in birds [58], and our results, even with a limited explanatory power of the model, tend to confirm that non-genetic factors could partly contribute to a wide range of traits.

### Possible inter and transgenerational transmission of genistein effect

The availability of phenotypic records for all individuals of the design highlighted an overall progressive increase of the epiline effect from G0 to G2 on BW. This phenomenon may be due to genetic drift [59, 60], even though the 'mirror' mating design was implemented to minimize genetic effects, ensuring a balanced origin of founders between epilines in each generation. David & Ricard [61] showed a potential impact of drift on the phenotypic difference observed between epilines after three generations on a simulated design similar to that used in our experiment. By running additional simulations across all generations of interest (Additional file 8 Figure S9) we show that the phenotypic difference observed between epilines (from G1 onwards for females and in G2 for males) in our experimental design appears greater than when simulating drift.

Alternatively, one could explain such change through an increasing environmental influence across generations. Recently, the impact of the parental epigenome on offspring’s developmental phenotypes has been a research topic of growing interest in different livestock species like cattle [62], pig [63], sheep [64] and poultry [65]. One might expect non-genetic (particularly epigenetic) differences between epilines to decrease progressively due to epigenetic reprogramming in the germline [66]. This resetting is not so clear in chicken regarding DNA methylation [67]. This may partly explain the observed multigenerational effect, as partial reprogramming may allow some epigenetic signatures to escape and be transmitted across generations [68].

Surprisingly, epigenetic effects can even strengthen over generations despite germline resetting. For example, ancestral vinclozolin exposure in rats seems to induce sperm methylation changes that persist beyond 13 generations, correlating with rising disease incidence [69]. Repeated exposures further amplify multigenerational adaptations, as seen in *C. elegans* [66, 70, 71] or already reported in quail [5]. Even without repeated challenges, effects may intensify via secondary genetic modifications as observed in rats treated with vinclozolin [72].

More generally, transgenerational studies conducted on different models reveal that environmental exposures leave epigenetic marks that are partially reset in the germline, but can yield progressive phenotypic changes through genetic or non-genetic mechanisms [66]. Within this framework, our observations may reflect gradual amplification of initial non-genetic perturbations in specific genomic or epigenomic regions, leading to an increase in the proportion of variance explained by epiline across generations, even post-exposure. Given the unclear transmission mechanism of environmental effects in birds, monitoring epigenetic changes throughout generations using molecular data would shed light on the molecular dynamics at play to verify this assumption in Japanese quails (*Coturnix japonica*).

Indeed, in this study, genistein was only administered once and individuals were not selected, suggesting a possible transmission of the environmental effects to the progeny. This hypothesis concurs with results found by Karami et al. [73] on the same dataset. Indeed, the authors designed an additional covariance for offspring descending from either epi + or epi- individuals, as they share an extra similarity due to their embryonic environment. The inclusion of this additional covariance was significantly different from 0 for BW traits, marking a probable transmission of genistein effects to the progeny. Their result supports a potential transmission of environmental effects via non-genetic mechanisms.

Indeed, several experimental studies show that genistein can modulate the epigenome, which offers a plausible mechanistic link between early exposure and multigenerational effects. Notably, genome-wide DNA methylation analyses in mouse embryonic stem cells revealed that genistein perturbed their methylation pattern, showing both regions that were more methylated and regions that were less methylated in genistein-treated cells than in control cells [13]. In a mouse breast cancer model, maternal genistein supplementation induced both hypo‑ and hyper‑methylated regions in the genomes of offspring, again pointing to a complex, locus‑specific action on DNA methylation [74].

These findings, together with broader evidence that DNA methylation and histone modifications can mediate multigenerational inheritance of environmental effects, motivated our focus on whether genistein-induced changes in the embryonic environment could have consequences that extend beyond the directly exposed generation.

## Conclusions

While the phenotypic effects of genistein exposure varied in nature and intensity across generations and traits, our findings demonstrate that an environmental exposure during development can induce persistent phenotypic changes, particularly on BW.

Further investigation is needed to understand fully the molecular mechanisms underlying the increased proportion of phenotypic variance explained by the epiline across generations. Although the contribution of genetic drift cannot be fully excluded despite the controlled mating design and the limitation of progeny artificial selection, a molecular analysis would help dissect the potential involvement of non-genetic inheritance mechanisms.

To better disentangle these effects, future studies will focus on dissecting genome-wide epigenetic profiling (e.g., DNA methylation) across generations. For the traits showing epiline effect, EWAS (epigenome wide association studies) and metQTL (methylation quantitative trait loci) analyses should be performed to identify genomic regions where epigenetic marks govern phenotypic changes or where genetic variation is driving epigenetic changes. This will help decipher the variance due to genetic and epigenetic mechanisms and molecular pathways involved in its inheritance.

## Supplementary Information

Below is the link to the electronic supplementary material.

Supplementary Material 1

Supplementary Material 2

Supplementary Material 3

Supplementary Material 4

Supplementary Material 5

Supplementary Material 6

Supplementary Material 7

Supplementary Material 8

## Data Availability

All raw data are available on Zenodo, 10.5281/zenodo.15796146 (https://zenodo.org/records/15796146). The code used to run the analysis is available on GitLab: https://forge.inrae.fr/stacy.rousse/transgenerational_pheno_quails. A previous version of this manuscript has been deposited as a preprint: 10.1101/2025.07.04.663137.

## References

[1] David I, Canario L, Combes S, Demars J. Intergenerational transmission of characters through genetics, epigenetics, microbiota, and learning in livestock. Front Genet. 2019;10:1058. 31737041 10.3389/fgene.2019.01058 PMC6834772

[2] Guerrero-Bosagna C, Morisson M, Liaubet L, Rodenburg TB, de Haas EN, Košťál Ľ, et al. Transgenerational epigenetic inheritance in birds. Environ Epigenet. 2018;4:dvy008. 29732172 10.1093/eep/dvy008 PMC5920295

[3] Holeski LM, Jander G, Agrawal AA. Transgenerational defense induction and epigenetic inheritance in plants. Trends Ecol Evol. 2012;27:618–26. 22940222 10.1016/j.tree.2012.07.011

[4] Skinner MK. What is an epigenetic transgenerational phenotype?: F3 or F2. Reprod Toxicol. 2008;25:2–6. 17949945 10.1016/j.reprotox.2007.09.001 PMC2249610

[5] Vitorino Carvalho A, Hennequet-Antier C, Rouger R, Delaveau J, Bordeau T, Crochet S, et al. Thermal conditioning of quail embryos has transgenerational and reversible long-term effects. J Anim Sci Biotechnol. 2023;14:124. 37784159 10.1186/s40104-023-00924-2 PMC10546792

[6] Leroux S, Gourichon D, Leterrier C, Labrune Y, Coustham V, Rivière S, et al. Embryonic environment and transgenerational effects in quail. Genet Sel Evol. 2017;49:14. 28125975 10.1186/s12711-017-0292-7 PMC5270212

[7] Charrier M, Lumineau S, George I, Meurisse M, Georgelin M, Palme R, et al. Maternal stress effects across generations in a precocial bird. R Soc Open Sci. 2024;11:231826. 39205998 10.1098/rsos.231826 PMC11349446

[8] Brun J-M, Bernadet M-D, Cornuez A, Leroux S, Bodin L, Basso B, et al. Influence of grand-mother diet on offspring performances through the male line in Muscovy duck. BMC Genet. 2015;16:145. 26690963 10.1186/s12863-015-0303-z PMC4687110

[9] Dolinoy DC, Weidman JR, Waterland RA, Jirtle RL. Maternal genistein alters coat color and protects Avy mouse offspring from obesity by modifying the fetal epigenome. Environ Health Perspect. 2006;114:567–72. 16581547 10.1289/ehp.8700 PMC1440782

[10] Duncan EJ, Gluckman PD, Dearden PK. Epigenetics, plasticity, and evolution: how do we link epigenetic change to phenotype? J Exp Zool B Mol Dev Evol. 2014;322:208–20. 24719220 10.1002/jez.b.22571

[11] Day JK, Bauer AM, desBordes C, Zhuang Y, Kim B-E, Newton LG, et al. Genistein alters methylation patterns in mice. J Nutr. 2002;132:2419S-S2423. 12163704 10.1093/jn/132.8.2419S

[12] Bilir B, Sharma NV, Lee J, Hammarstrom B, Svindland A, Kucuk O, et al. Effects of genistein supplementation on genome-wide DNA methylation and gene expression in patients with localized prostate cancer. Int J Oncol. 2017;51:223–34. 28560383 10.3892/ijo.2017.4017 PMC5467777

[13] Sato N, Yamakawa N, Masuda M, Sudo K, Hatada I, Muramatsu M. Genome-wide DNA methylation analysis reveals phytoestrogen modification of promoter methylation patterns during embryonic stem cell differentiation. PLoS ONE. 2011;6:e19278. 21559447 10.1371/journal.pone.0019278 PMC3084807

[14] Grgic D, Varga E, Novak B, Müller A, Marko D. Isoflavones in animals: metabolism and effects in livestock and occurrence in feed. Toxins (Basel). 2021;13:836. 34941674 10.3390/toxins13120836 PMC8705642

[15] Cerutti C, Leroux S, Terzian P, Lledo J, Gourichon D, Hubert J-N, et al. Transgenerational epigenetics in quail: whole genome DNA methylation analysis. ETH Zurich, Switzerland. 2023. https://hal.inrae.fr/hal-04212971. Accessed 15 Apr 2026.

[16] Bonduriansky R, Day T. Nongenetic inheritance and its evolutionary implications. Annu Rev Ecol Evol Syst. 2009;40:103–25.

[17] Day T, Bonduriansky R. A unified approach to the evolutionary consequences of genetic and nongenetic inheritance. Am Nat. 2011;178:E18-36. 21750377 10.1086/660911

[18] Onderci M, Sahin K, Sahin N, Gursu MF, Doerge D, Sarkar FH, et al. The effect of genistein supplementation on performance and antioxidant status of Japanese quail under heat stress. Arch Anim Nutr. 2004. 10.1080/00039420400020017. 15732579 10.1080/00039420400020017

[19] Chomchuen K, Tuntiyasawasdikul V, Chankitisakul V, Boonkum W. Genetic evaluation of body weights and egg production traits using a multi-trait animal model and selection index in Thai native synthetic chickens (Kaimook e-san2 ). Animals (Basel). 2022;12:335. 35158657 10.3390/ani12030335 PMC8833322

[20] Gaya LG, Ferraz JBS, Rezende FM, Mourão GB, Mattos EC, Eler JP, et al. Heritability and genetic correlation estimates for performance and carcass and body composition traits in a male broiler line. Poult Sci. 2006;85:837–43. 16673760 10.1093/ps/85.5.837

[21] Lormant F, Ferreira VHB, Meurisse M, Lemarchand J, Constantin P, Morisse M, et al. Emotionality modulates the impact of chronic stress on memory and neurogenesis in birds. Sci Rep. 2020;10:14620. 32884096 10.1038/s41598-020-71680-w PMC7471904

[22] Prince LLL, Rajaravindra KS, Rajkumar U, Reddy BLN, Paswan C, Haunshi S, et al. Genetic analysis of growth and egg production traits in synthetic colored broiler female line using animal model. Trop Anim Health Prod. 2020;52:3153–63. 32617799 10.1007/s11250-020-02340-4

[23] van Oers K, Drent PJ, de Goede P, van Noordwijk AJ. Realized heritability and repeatability of risk-taking behaviour in relation to avian personalities. Proc R Soc Lond B Biol Sci. 2004;271:65–73. 10.1098/rspb.2003.2518 PMC1691563 15002773

[24] Wright D, Henriksen R. Advances in understanding the genetics of poultry behaviour. In: Understanding the behaviour and improving the welfare of chickens. 2020. https://api.semanticscholar.org/CorpusID:224989248.

[25] Mills AD, Faure J-M. Divergent selection for duration of tonic immobility and social reinstatement behavior in Japanese quail (*Coturnix coturnix japonica*) chicks. J Comp Psychol. 1991;105:25–38. 2032452 10.1037/0735-7036.105.1.25

[26] Komsta L, Novomestky F. moments: moments, cumulants, skewness, kurtosis and related tests. 2022. 10.32614/CRAN.package.moments.

[27] Fink EL. The FAQs on data transformation. Commun Monogr. 2009;76:379–97.

[28] Venables WN, Ripley BD. Modern applied statistics with S. 4th ed. New York: Springer; 2002.

[29] Lê S, Josse J, Husson F. FactoMineR: a package for multivariate analysis. J Stat Softw. 2008;25:1–18.

[30] R Core Team. R: a language and environment for statistical computing. Vienna: R Foundation for Statistical Computing. 2025. https://www.R-project.org/.

[31] Rockwood S, Mason D, Lord R, Lamar P, Prozialeck W, Al-Nakkash L. Genistein diet improves body weight, serum glucose and triglyceride levels in both male and female ob/ob mice. Diabetes Metab Syndr Obes. 2019;12:2011–21. 31686880 10.2147/DMSO.S216312 PMC6783398

[32] Xiang J, Mlambo R, Dube P, Machona O, Shaw I, Seid Y, et al. The obesogenic side of genistein. Front Endocrinol (Lausanne ). 2023;14:1308341. 38098865 10.3389/fendo.2023.1308341 PMC10720314

[33] Robinson FE, Wilson JL, Yu MW, Fasenko GM, Hardin RT. The relationship between body weight and reproductive efficiency in meat-type chickens. Poult Sci. 1993;72:912–22.

[34] Mahmoud Ali F, Hamid HK. Epigenetic effects of selenium and vitamin E supplementation in broiler breeder diets on the performance of their progeny. RB. 2022;7:1–5.

[35] Aldahmani H, Rasah M. Different stages of poultry production require specific nutrient profiles. AlQalam J Med Appl Sci. 2026;45:15.

[36] Lin F, Wu J, Abdelnabi MA, Ottinger MA, Giusti MM. Effects of dose and glycosylation on the transfer of genistein into the eggs of the Japanese quail (*Coturnix japonica*). J Agric Food Chem. 2004;52:2397–403. 15080653 10.1021/jf034921f

[37] Casari G, Romaldi B, Scirè A, Minnelli C, Marzioni D, Ferretti G, et al. Epigenetic properties of compounds contained in functional foods against cancer. Biomolecules. 2025;15:15. 10.3390/biom15010015 PMC11762081 39858410

[38] Rasheed S, Rehman K, Shahid M, Suhail S, Akash MSH. Therapeutic potentials of genistein: new insights and perspectives. J Food Biochem. 2022;46:e14228. 35579327 10.1111/jfbc.14228

[39] Liu X, Li F, Xie J, Huang D, Xie M. Fetal and neonatal genistein exposure aggravates to interfere with ovarian follicle development of obese female mice induced by high-fat diet. Food Chem Toxicol. 2020;135:110982. 31747621 10.1016/j.fct.2019.110982

[40] Lozi AA, Pinto da Matta SL, Sarandy MM, Silveira Alves de Melo FC, Araujo DC, Novaes RD, et al. Relevance of the isoflavone absorption and testicular function: a systematic review of preclinical evidence. Evid Based Complement Alternat Med. 2021. 10.1155/2021/8853172. 33628321 10.1155/2021/8853172 PMC7895610

[41] Rashid R, Kumari A, Chattopadhyay N, Jha R, Rajender S. Genistein lowers fertility with pronounced effect in males: meta-analyses on pre-clinical studies. Andrologia. 2022;54:e14511. 35760341 10.1111/and.14511

[42] Toktay E, Selli J, Gurbuz MA, Tastan TB, Ugan RA, Un H, et al. Effects of soy isoflavonoids (genistein and daidzein) on endometrial receptivity. Iran J Basic Med Sci. 2020;23:1603–9. 33489035 10.22038/ijbms.2020.48294.11089 PMC7811811

[43] Svensson T, Sawada N, Mimura M, Nozaki S, Shikimoto R, Tsugane S. Midlife intakes of the isoflavone genistein and soy and the risk of late-life cognitive impairment: the JPHC Saku mental health study. J Epidemiol. 2023;33:342–9. 34924453 10.2188/jea.JE20210199 PMC10257986

[44] Brulport A, Le Corre L, Maquart G, Barbet V, Dastugue A, Severin I, et al. Multigenerational study of the obesogen effects of bisphenol S after a perinatal exposure in C57BL6/J mice fed a high fat diet. Environ Pollut. 2021;270:116243. 33326921 10.1016/j.envpol.2020.116243

[45] Naponelli V, Piscazzi A, Mangieri D. Cellular and molecular mechanisms modulated by genistein in cancer. Int J Mol Sci. 2025;26:1114. 39940882 10.3390/ijms26031114 PMC11818640

[46] Sharifi-Rad J, Quispe C, Imran M, Rauf A, Nadeem M, Gondal TA, et al. Genistein: an integrative overview of its mode of action, pharmacological properties, and health benefits. Oxid Med Cell Longev. 2021;2021:3268136. 34336089 10.1155/2021/3268136 PMC8315847

[47] Ross JA, Kasum CM. Dietary flavonoids: bioavailability, metabolic effects, and safety. Annu Rev Nutr. 2002;22:19–34. 12055336 10.1146/annurev.nutr.22.111401.144957

[48] Kamboh AA, Zhu W. Individual and combined effects of genistein and hesperidin supplementation on meat quality in meat‐type broiler chickens. J Sci Food Agric. 2013;93:3362–7. 23605817 10.1002/jsfa.6185

[49] Rasouli E, Jahanian R. Improved performance and immunological responses as the result of dietary genistein supplementation of broiler chicks. Animal (Basel). 2015;9:1473–80. 10.1017/S1751731115000853 25998982

[50] Lv Z, Fan H, Zhang B, Ning C, Xing K, Guo Y. Dietary genistein supplementation in laying broiler breeder hens alters the development and metabolism of offspring embryos as revealed by hepatic transcriptome analysis. FASEB J. 2018;32:4214–28. 29518347 10.1096/fj.201701457R

[51] Cerutti C, Leroux S, Gourichon D, Labrune Y, David I, Zerjal T, et al. Short communication: effects of in-ovo injection of endocrine disruptors and methyltransferase inhibitor on quail growth and egg-laying performances. Animal (Basel). 2022;16:100464. 10.1016/j.animal.2022.100464 35180683

[52] Akdemir F, Sahin K. Genistein supplementation to the quail: effects on egg production and egg yolk genistein, daidzein, and lipid peroxidation levels. Poult Sci. 2009;88:2125–31. 19762866 10.3382/ps.2009-00004

[53] Navarro-Guillén C, Huesa-Cerdán R, Hidalgo-Pérez JA, Simó-Mirabet P, Rodríguez-Viera L, Martos-Sitcha JA, et al. One-carbon nutrients and genistein as nutritional programming effectors in juvenile gilthead seabream (*Sparus aurata*): contrasting effects on phenotypic traits. Aquaculture. 2025;598:742063.

[54] Halbersleben DL, Mussehl FE. Relation of egg weight to chick weight at hatching. Poult Sci. 1922;1:143–4.

[55] Widowski TM, Cooley L, Hendriksen S, Peixoto MRLV. Maternal age and maternal environment affect egg composition, yolk testosterone, offspring growth and behaviour in laying hens. Sci Rep. 2022;12:1828. 35115547 10.1038/s41598-022-05491-6 PMC8814016

[56] Rutschmann A, de Villemereuil P, Brekke P, Ewen JG, Anderson N, Santure AW. Consequences of space sharing on individual phenotypes in the New Zealand hihi. Evol Ecol. 2020;34:821–39.

[57] Béziers P, San-Jose LM, Almasi B, Jenni L, Roulin A. Baseline and stress-induced corticosterone levels are heritable and genetically correlated in a barn owl population. Heredity (Edinb). 2019;123:337–48. 30837668 10.1038/s41437-019-0203-5 PMC6781159

[58] Frésard L, Morisson M, Brun J-M, Collin A, Pain B, Minvielle F, et al. Epigenetics and phenotypic variability: some interesting insights from birds. Genet Sel Evol. 2013;45:16. 23758635 10.1186/1297-9686-45-16 PMC3693910

[59] Falconer DS, Mackay TFC. Introduction to quantitative genetics. 4th ed. Harlow, Essex, UK: Longman; 1996.

[60] Hospital F, Chevalet C. Interactions of selection, linkage and drift in the dynamics of polygenic characters. Genet Res. 1996;67:77–87. 8919890 10.1017/s0016672300033498

[61] David I, Ricard A. An improved transmissibility model to detect transgenerational transmitted environmental effects. Genet Sel Evol. 2023;55:66. 37735633 10.1186/s12711-023-00833-y PMC10512618

[62] Kiefer H, Sellem E, Bonnet-Garnier A, Pannetier M, Costes V, Schibler L, et al. The epigenome of male germ cells and the programming of phenotypes in cattle. Anim Front. 2021;11:28–38. 34934527 10.1093/af/vfab062 PMC8683155

[63] Wu P, Wang J, Ji X, Chai J, Chen L, Zhang T, et al. Maternal hypermethylated genes contribute to intrauterine growth retardation of piglets in Rongchang pigs. Int J Mol Sci. 2024;25:6462. 38928167 10.3390/ijms25126462 PMC11203632

[64] Fonseca PAS, Suárez-Vega A, Pelayo R, Marina H, Alonso-García M, Gutiérrez-Gil B, et al. Intergenerational impact of dietary protein restriction in dairy ewes on epigenetic marks in the perirenal fat of their suckling lambs. Sci Rep. 2023;13:4351. 36928446 10.1038/s41598-023-31546-3 PMC10020577

[65] Hu Y, Feng Y, Ding Z, Lv L, Sui Y, Sun Q, et al. Maternal betaine supplementation decreases hepatic cholesterol deposition in chicken offspring with epigenetic modulation of SREBP2 and CYP7A1 genes. Poult Sci. 2020;99:3111–20. 32475448 10.1016/j.psj.2019.12.058 PMC7597551

[66] Burton NO, Greer EL. Multigenerational epigenetic inheritance: transmitting information across generations. Semin Cell Dev Biol. 2022;127:121–32. 34426067 10.1016/j.semcdb.2021.08.006 PMC8858334

[67] Kress C, Jouneau L, Pain B. Reinforcement of repressive marks in the chicken primordial germ cell epigenetic signature: divergence from basal state resetting in mammals. Epigenetics Chromatin. 2024;17:11. 38671530 10.1186/s13072-024-00537-7 PMC11046797

[68] Heard E, Martienssen RA. Transgenerational epigenetic inheritance: myths and mechanisms. Cell. 2014;157:95–109. 24679529 10.1016/j.cell.2014.02.045 PMC4020004

[69] Korolenko AA, Nilsson EE, De Santos S, Skinner MK. Generational stability of environmentally induced epigenetic transgenerational inheritance of adult-onset disease over ten mammalian generations. Environ Epigenet. 2025;11:dvaf033. 41438350 10.1093/eep/dvaf033 PMC12720976

[70] Burton NO, Riccio C, Dallaire A, Price J, Jenkins B, Koulman A, et al. Cysteine synthases CYSL-1 and CYSL-2 mediate C. elegans heritable adaptation to P. vranovensis infection. Nat Commun. 2020;11:1741. 32269224 10.1038/s41467-020-15555-8 PMC7142082

[71] Liberman N, Wang SY, Greer EL. Transgenerational epigenetic inheritance: from phenomena to molecular mechanisms. Curr Opin Neurobiol. 2019;59:189–206. 31634674 10.1016/j.conb.2019.09.012 PMC6889819

[72] Skinner MK, Guerrero-Bosagna C, Haque MM. Environmentally induced epigenetic transgenerational inheritance of sperm epimutations promote genetic mutations. Epigenetics. 2015;10:762–71. 26237076 10.1080/15592294.2015.1062207 PMC4622673

[73] Karami K, Rousse S, Pitel F, Eynard S, Gourichon D, Leroux S, et al. Transmissibility model to evaluate transgenerational transmission of environmental effects in quails. Animal. 2025;19:101636. 41004862 10.1016/j.animal.2025.101636

[74] Chen M, Li S, Srinivasasainagendra V, Sharma M, Li Z, Tiwari H, et al. Maternal soybean genistein on prevention of later-life breast cancer through inherited epigenetic regulations. Carcinogenesis. 2022;43:190–202. 35084457 10.1093/carcin/bgac009 PMC9036993

[75] Faux A-M, Gorjanc G, Gaynor RC, Battagin M, Edwards SM, Wilson DL, et al. AlphaSim: software for breeding program simulation. Plant Genome. 2016;9:14. 10.3835/plantgenome2016.02.0013 27902803

[76] Gaynor RC, Gorjanc G, Hickey JM. AlphaSimR an R package for breeding program simulations. G3 (Bethesda). 2020;11:jkaa017. 10.1093/g3journal/jkaa017 PMC8022926 33704430

