# Supplementary material for: Transmission of an environmental modification in quails across three generations: changes in the contribution to phenotypic variability
Source: Genet Sel Evol. 2026 Aug 10;58:57. doi: 10.1186/s12711-026-01079-0 (PMC13501780; doi:10.1186/s12711-026-01079-0)
Supplement: Supplementary file 2 [file 12711_2026_1079_MOESM2_ESM.pdf]

Additional file 2

Table S1 - Summary table of Shapiro-Wilk normality tests in each sex and generation. The Null Hypothesis tested is H0 "The distribution of the data does not deviate from a normal distribution" at a significance threshold of 0.05. FDR corrections to account for multiple testing were performed and corresponds to the p adj column. Shapiro-Wilk normality tests confirm that a variety of traits deviate from normality.

**Trait**	**generation**	**sex**	**pval**	**test**	**p adj**
bw_1w	Gen0	male	0.0055	0.9814	0.0089
bw_4w	Gen0	male	0.05556	0.98766	0.0765
bw_7w	Gen0	male	0.14311	0.99018	0.1856
bw_sl	Gen0	male	0.92455	0.99644	0.9322
adj_heartW	Gen0	male	0.00004	0.79872	$8.71e^{-5}$
adj_livrW	Gen0	male	0.16773	0.94987	0.2088
adj_adipW	Gen0	male	0	0.93188	0
centreDuration	Gen0	male	0	0.95049	0
periphFreq	Gen0	male	0	0.90406	0
totalDist	Gen0	male	0.00003	0.96234	$7.47e^{-5}$
bw_1w	Gen0	female	0.01912	0.98443	0.0281
bw_4w	Gen0	female	0.14607	0.98999	0.1856
bw_7w	Gen0	female	0.35133	0.99248	0.4082
bw_sl	Gen0	female	0.51931	0.99332	0.5812
adj_heartW	Gen0	female	0.00004	0.93073	$8.71e^{-5}$
adj_livrW	Gen0	female	0.00131	0.96326	0.0024
adj_adipW	Gen0	female	0	0.94621	0
centreDuration	Gen0	female	0	0.94859	0
periphFreq	Gen0	female	0	0.90435	0
totalDist	Gen0	female	0	0.92232	0
LR	Gen0	female	0	0.74957	0

adj_EW	Gen0	female	0.57197	0.99346	0.6180
precocity	Gen0	female	0	0.70465	0
bw_1w	Gen1	male	0.00356	0.9769	0.0059
bw_4w	Gen1	male	0.01128	0.98078	0.0174
bw_7w	Gen1	male	0.5876	0.99352	0.6180
bw_sl	Gen1	male	0.34441	0.99112	0.4079
adj_heartW	Gen1	male	0.00354	0.97542	0.0059
adj_livrW	Gen1	male	0	0.89834	0
adj_adipW	Gen1	male	0	0.80282	0
adj_testisW	Gen1	male	0.00001	0.94869	2.71e-5
centreDuration	Gen1	male	0.00003	0.95721	7.47e-5
periphFreq	Gen1	male	0.00004	0.96003	8.71e-5
totalDist	Gen1	male	0.00091	0.97188	0.0017
bw_1w	Gen1	female	0.0003	0.97109	0.0006
bw_4w	Gen1	female	0.10728	0.98881	0.1423
bw_7w	Gen1	female	0.60532	0.99418	0.6258
bw_sl	Gen1	female	0.58107	0.99376	0.6180
adj_heartW	Gen1	female	0.49325	0.99313	0.5624
adj_livrW	Gen1	female	0.03181	0.98471	0.046
adj_adipW	Gen1	female	0	0.86057	0
centreDuration	Gen1	female	0	0.93779	0
periphFreq	Gen1	female	0	0.93923	0
totalDist	Gen1	female	0	0.9475	0
LR	Gen1	female	0	0.8038	0
adj_EW	Gen1	female	0.00018	0.96668	0.00037
precocity	Gen1	female	0	0.81536	0
bw_1w	Gen2	male	0.0136	0.98674	0.0205

bw_4w	Gen2	male	0.24305	0.99308	0.2936
bw_7w	Gen2	male	0.01044	0.98605	0.0165
bw_sl	Gen2	male	0.05579	0.98714	0.0765
adj_heartW	Gen2	male	0	0.76497	0
adj_livrW	Gen2	male	0.00004	0.96431	8.71e-5
adj_adipW	Gen2	male	0	0.83689	0
adj_testisW	Gen2	male	0.95259	0.99685	0.9526
centreDuration	Gen2	male	0.00274	0.97748	0.00485
periphFreq	Gen2	male	0	0.87496	0
totalDist	Gen2	male	0.0004	0.97161	0.00079
bw_1w	Gen2	female	0.00247	0.98148	0.00443
bw_4w	Gen2	female	0.53771	0.99457	0.5964
bw_7w	Gen2	female	0.23206	0.99217	0.2860
bw_sl	Gen2	female	0.08	0.98793	0.1084
adj_heartW	Gen2	female	0.00006	0.96436	0.00012
adj_livrW	Gen2	female	0.69729	0.99467	0.7149
adj_adipW	Gen2	female	0	0.90878	0
centreDuration	Gen2	female	0	0.94936	0
periphFreq	Gen2	female	0	0.92953	0
totalDist	Gen2	female	0	0.95454	0
LR	Gen2	female	0	0.81864	0
adj_EW	Gen2	female	0.00183	0.97618	0.0033
precocity	Gen2	female	0	0.72279	0

Table S2 - Summary table of Kruskal-Wallis mean comparisons between epilines for each sex and generation. The Null Hypothesis tested is H0 “There is no difference between epilines” at a significance threshold of 0.05. FDR corrections to account for multiple testing were performed and corresponds to the p adj column. Kruskal-Wallis tests evidence mean differences between epilines for BW from G1 onwards in females, and in G2 males.

**Trait**	**generation**	**sex**	**pval**	**test**	**p adj**
bw_1w	Gen0	male	0.68216	0.1677	0.8969
bw_4w	Gen0	male	0.09981	2.70853	0.2812
bw_7w	Gen0	male	0.33577	0.92652	0.6003
bw_sl	Gen0	male	0.17709	1.82187	0.4191
adj_heartW	Gen0	male	0.93991	0.00568	0.9753
adj_livrW	Gen0	male	0.96691	0.00172	0.9753
adj_adipW	Gen0	male	0.16464	1.9311	0.4133
centreDuration	Gen0	male	0.25636	1.2883	0.5322
periphFreq	Gen0	male	0.58415	0.29957	0.8585
totalDist	Gen0	male	0.80374	0.06176	0.9233
bw_1w	Gen0	female	0.35732	0.84729	0.6188
bw_4w	Gen0	female	0.09673	2.75863	0.2812
bw_7w	Gen0	female	0.41576	0.66227	0.6676
bw_sl	Gen0	female	0.12459	2.35866	0.3276
adj_heartW	Gen0	female	0.64952	0.2065	0.8727
adj_livrW	Gen0	female	0.73145	0.11778	0.9233
adj_adipW	Gen0	female	0.37307	0.79343	0.6307
centreDuration	Gen0	female	0.26985	1.21749	0.5322
periphFreq	Gen0	female	0.83123	0.04542	0.9233
totalDist	Gen0	female	0.83226	0.04486	0.9233
LR	Gen0	female	0.86775	0.02773	0.9335
adj_EW	Gen0	female	0.10298	2.65884	0.2812

precocity	Gen0	female	0.78207	0.07652	0.9233
bw_1w	Gen1	male	0.86248	0.03001	0.9335
bw_4w	Gen1	male	0.05526	3.67414	0.1966
bw_7w	Gen1	male	0.05538	3.67046	0.1966
bw_sl	Gen1	male	0.30982	1.03143	0.5789
adj_heartW	Gen1	male	0.00006	16.22683	0.00047
adj_livrW	Gen1	male	0.6167	0.25053	0.8585
adj_adipW	Gen1	male	0.80153	0.06319	0.9233
adj_testisW	Gen1	male	0.40768	0.68557	0.6676
centreDuration	Gen1	male	0.03149	4.62593	0.1294
periphFreq	Gen1	male	0.74946	0.10198	0.9233
totalDist	Gen1	male	0.42314	0.64157	0.6676
bw_1w	Gen1	female	0.00001	19.96487	0.000089
bw_4w	Gen1	female	0.00007	15.70274	0.000497
bw_7w	Gen1	female	0.00522	7.80164	0.02850
bw_sl	Gen1	female	0.00311	8.74096	0.01840
adj_heartW	Gen1	female	0.08176	3.02952	0.2639
adj_livrW	Gen1	female	0.21855	1.51386	0.4849
adj_adipW	Gen1	female	0.16883	1.89332	0.4133
centreDuration	Gen1	female	0.79135	0.06999	0.9233
periphFreq	Gen1	female	0.22805	1.453	0.4907
totalDist	Gen1	female	0.7718	0.08411	0.9233
LR	Gen1	female	0.96137	0.00235	0.9753
adj_EW	Gen1	female	0.60537	0.26697	0.8585
precocity	Gen1	female	0.00792	7.05164	0.0402
bw_1w	Gen2	male	0.00022	13.63858	0.0014
bw_4w	Gen2	male	0	38.18889	$< 1e^{-6}$

bw_7w	Gen2	male	0	24.14779	$< 1e^{-6}$
bw_sl	Gen2	male	0.00001	19.06475	0.000089
adj_heartW	Gen2	male	0.00868	6.88696	0.0411
adj_livrW	Gen2	male	0.07319	3.21006	0.2475
adj_adipW	Gen2	male	0.2646	1.24451	0.5322
adj_testisW	Gen2	male	0.18355	1.76867	0.4204
centreDuration	Gen2	male	0.4461	0.58055	0.6885
periphFreq	Gen2	male	0.0241	5.08731	0.1069
totalDist	Gen2	male	0.65145	0.20408	0.8727
bw_1w	Gen2	female	0	25.20151	$< 1e^{-6}$
bw_4w	Gen2	female	0	41.65775	$< 1e^{-6}$
bw_7w	Gen2	female	0	31.61942	$< 1e^{-6}$
bw_sl	Gen2	female	0	29.34427	$< 1e^{-6}$
adj_heartW	Gen2	female	0.55856	0.34221	0.8438
adj_livrW	Gen2	female	0.30237	1.06371	0.5789
adj_adipW	Gen2	female	0.10166	2.67932	0.2812
centreDuration	Gen2	female	0.33818	0.91732	0.6003
periphFreq	Gen2	female	0.60951	0.26089	0.8585
totalDist	Gen2	female	0.97526	0.00096	0.9753
LR	Gen2	female	0.0328	4.55607	0.1294
adj_EW	Gen2	female	0.83055	0.0458	0.9233
precocity	Gen2	female	0.89131	0.01867	0.9445

Table S3 - Summary table of applied transformations for normalization and skewness values. raw: no transformation, log10: Log10 transformation, sqrt: square root transformation, inv: inverse transformation, best: transformation chosen that minimizes data skewness.
bw_1w: bodyweight at one week, bw_4w: bodyweight at four weeks, bw_7w: bodyweight at seven weeks, bw_sl: bodyweight at slaughter, adj_heartW: adjusted heart weight, adj_livrW: adjusted liver weight, adj_adipW: adjusted abdominal fat weight, LR: laying rate, adj_EW: adjusted egg weight, totalDist: total distance, centreDuration: duration at centre, periphFreq: frequency at periphery.

**Trait**	**raw**	**log10**	**sqrt**	**inv**	**best**
bw_1w	0.010	0.483	0.219	1.211	raw
bw_4w	0.071	0.373	0.218	0.717	raw
bw_7w	0.151	0.051	0.050	0.256	sqrt
bw_sl	0.109	0.112	0.0002	0.347	sqrt
adj_heartW	1.552	0.382	0.522	3.241	log10
adj_livrW	0.250	0.322	0.038	0.953	sqrt
adj_adipW	1.572	1.535	0.159	1.500	sqrt
adj_testisW	0.438	4.472	1.607	15.976	raw
LR	1.916	4.408	2.767	10.605	raw
precocity	2.137	1.794	1.961	1.481	inv
adj_EW	0.245	0.248	0.003	0.802	sqrt
totalDist	0.910	1.071	0.012	4.437	sqrt
centreDuration	0.374	2.085	0.323	21.861	sqrt
periphFreq	1.691	0.853	0.107	2.886	sqrt
