# Supplementary material for: Transmission of an environmental modification in quails across three generations: changes in the contribution to phenotypic variability
Source: Genet Sel Evol. 2026 Aug 10;58:57. doi: 10.1186/s12711-026-01079-0 (PMC13501780; doi:10.1186/s12711-026-01079-0)
Supplement: Supplementary file 3 [file 12711_2026_1079_MOESM3_ESM.pdf]

Additional file 3: Contribution of parameters to phenotypic variability per generation.

Table S4 - Summary table of the linear model fitted for each trait with the best set of parameters kept after the stepAIC. The “trans” suffix was added when the trait was transformed during the normalization step. bw_1w: bodyweight at one week, bw_4w: bodyweight at four weeks, bw_7w: bodyweight at seven weeks, bw_sl: bodyweight at slaughter, adj_heartW: adjusted heart weight, adj_livrW: adjusted liver weight, adj_adipW: adjusted abdominal fat weight, LR: laying rate, adj_EW: adjusted egg weight, totalDist: total distance, centreDuration: duration at centre, periphFreq: frequency at periphery.

**Generation**	**Trait**	**Linear model equation**
G0	bw_1w	trait ~ sex + family
G1	bw_1w	trait ~ sex + epiline + family + sex:epiline
G2	bw_1w	trait ~ sex + epiline + family + epiline:family
G0	bw_4w	trait ~ sex + epiline + family + epiline:family
G1	bw_4w	trait ~ sex + epiline + family
G2	bw_4w	trait ~ sex + epiline + family + epiline:family
G0	bw_7w_trans	trait ~ sex + epiline + family + epiline:family
G1	bw_7w_trans	trait ~ sex + epiline + family + epiline:family
G2	bw_7w_trans	trait ~ sex + epiline + family + epiline:family
G0	bw_sl_trans	trait ~ sex + epiline + family + epiline:family + sex:epiline
G1	bw_sl_trans	trait ~ sex + epiline + family + epiline:family
G2	bw_sl_trans	trait ~ sex + epiline + family + epiline:family
G0	adj_heartW_trans	trait ~ sex + epiline + repro + sex:epiline + sex:repro
G1	adj_heartW_trans	trait ~ sex + epiline + family + repro + epiline:family + epiline:repro
G2	adj_heartW_trans	trait ~ sex + epiline + family + sex:epiline
G0	adj_livrW_trans	trait ~ sex + family + repro + sex:repro
G1	adj_livrW_trans	trait ~ sex + repro + sex:repro
G2	adj_livrW_trans	trait ~ sex + epiline + sex:epiline
G0	adj_adipW_trans	trait ~ sex + epiline + family + repro + sex:epiline + epiline:repro + sex:repro
G1	adj_adipW_trans	trait ~ sex + epiline + family + repro + epiline:family + epiline:repro + sex:repro
G2	adj_adipW_trans	trait ~ sex + epiline + family + repro + epiline:family + epiline:repro

G1	adj_testisW	trait ~ epiline + repro + epiline:repro
G2	adj_testisW	trait ~ epiline + family + epiline:family
G0	LR	trait ~ repro
G1	LR	trait ~ family + repro
G2	LR	trait ~ family
G0	precocity_trans	trait ~ epiline + family + epiline:family
G1	precocity_trans	trait ~ epiline
G2	precocity_trans	trait ~ epiline + family
G0	adj_EW_trans	trait ~ epiline + family + repro + epiline:family + epiline:repro
G1	adj_EW_trans	trait ~ epiline + repro + epiline:repro
G2	adj_EW_trans	trait ~ epiline + family + epiline:family
G0	totalDist_trans	trait ~ sex + family + repro + sex:repro
G1	totalDist_trans	trait ~ sex + epiline + family + repro + sex:epiline + sex:repro
G2	totalDist_trans	trait ~ sex + epiline + family + epiline:family
G0	centreDuration_trans	trait ~ sex + epiline + repro + sex:repro
G1	centreDuration_trans	trait ~ sex + epiline + repro + sex:epiline + epiline:repro
G2	centreDuration_trans	trait ~ repro
G0	periphFreq_trans	trait ~ sex + family + repro + sex:repro
G1	periphFreq_trans	trait ~ sex + epiline + family
G2	periphFreq_trans	trait ~ sex + epiline

Table S5 - Summary table of the estimates of the contribution of parameters (and standard deviation) to phenotypic variability (expressed in percentage) for tissue weights, production and behavioural traits.

Trait	gen	epiline	family	sex	repro	interaction epi	interaction non epi	residuals
adj. abdo fat	G0	0.14 (0.14)	10.04 (1.13)	10.54 (1 .22)	11.43 (1.11)	0.8 (0.33)	8.69 (0.93)	58.37 (3.12)
adj. abdo fat	G1	0.05 (0.07)	3.75 (0.8)	16.57 (1.26)	15.53 (1.08)	6.75 (1.01)	14.64 (0.92)	42.71 (2.17)
adj. abdo fat	G2	0.79 (0.21)	9.43 (1.61)	10.22 (1.47)	0.21 (0.13)	10.86 (1.49)	0 (0)	68.5 (2.24)
adj. heart weight	G0	1.91 (1.13)	0 (0)	0.34 (0.22)	1.45 (0.61)	2.25 (1.21)	1.84 (0.75)	92.21 (2.98)
adj. heart weight	G1	0.58 (0.36)	7.03 (1.15)	24.05 (2.12)	0.41 (0.26)	11.53 (1.4)	0 (0)	56.41 (2.05)
adj. heart weight	G2	1.42 (0.59)	12.36 (1.51)	11.72 (1.54)	0 (0)	1.18 (0.56)	0 (0)	73.32 (2.14)
adj. liver weight	G0	0 (0)	26.87 (3.04)	4.55 (1.73)	0.28 (0.19)	0 (0)	0.57 (0.31)	67.74 (2.95)
adj. liver weight	G1	0 (0)	0 (0)	17.3 (2.49)	4.14 (0.88)	0 (0)	3.59 (0.83)	74.96 (1.7)
adj. liver weight	G2	0.3 (0.17)	0 (0)	66.57 (1.23)	0 (0)	0.39 (0.19)	0 (0)	32.74 (1.32)
adj. testicle weight	G1	2.15 (1.48)	0 (0)	-	7.34 (2.19)	2.01 (1.45)	0 (0)	88.49 (3.34)
adj. testicle weight	G2	0.27 (0.43)	10.67 (2.57)	-	0 (0)	18.08 (3.53)	0 (0)	70.99 (3.77)
LR	G0	0 (0)	0 (0)	-	7.02 (1.89)	0 (0)	0 (0)	92.98 (1.89)
LR	G1	0 (0)	17.13 (3.86)	-	4.63 (2.24)	0 (0)	0 (0)	78.24 (4.14)
LR	G2	0 (0)	15.38 (4.67)	-	0 (0)	0 (0)	0 (0)	84.62 (4.67)
adj. EW	G0	4.79 (2.15)	37.6 (4.22)	-	0.28 (0.31)	13.13 (2.83)	0 (0)	44.2 (4.26)
adj. EW	G1	4.95 (2.73)	0 (0)	-	0.5 (0.68)	4.36 (2.18)	0 (0)	90.18 (5.07)
adj. EW	G2	0.87	23.91	-	0	16.44	0	58.79

		(0.94)	(3.39)		(0)	(3.38)	(0)	(5.05)
precocity	G0	0.5 (0.66)	19.31 (3.63)	-	0 (0)	18.41 (2.84)	0 (0)	61.78 (4.82)
precocity	G1	1.54 (1.25)	0 (0)	-	0 (0)	0 (0)	0 (0)	98.46 (1.25)
precocity	G2	0.42 (0.61)	11.28 (3.39)	-	0 (0)	0 (0)	0 (0)	88.3 (3.48)
duration at centre	G0	0.57 (0.34)	0 (0)	0.76 (0.43)	1.61 (0.59)	0 (0)	0.84 (0.44)	96.22 (1.31)
duration at centre	G1	0.47 (0.38)	0 (0)	0.78 (0.47)	0.17 (0.2)	1.59 (0.73)	0 (0)	96.99 (1.12)
duration at centre	G2	0 (0)	0 (0)	0 (0)	0.72 (0.21)	0 (0)	0 (0)	99.28 (0.21)
freq. at periphery	G0	0 (0)	10.45 (1.45)	9.41 (1.23)	0.57 (0.33)	0 (0)	0.92 (0.43)	78.65 (2.2)
freq. at periphery	G1	0.68 (0.43)	11.98 (1.46)	9.99 (1.4)	0 (0)	0 (0)	0 (0)	77.35 (2.12)
freq. at periphery	G2	0.57 (0.4)	0 (0)	4.98 (0.95)	0 (0)	0 (0)	0 (0)	94.46 (1.02)
total distance	G0	0 (0)	9.68 (1.27)	18.46 (1.66)	1.13 (0.46)	0 (0)	1.43 (0.51)	69.3 (2.11)
total distance	G1	0.61 (0.39)	14.55 (1.74)	4.77 (1.14)	0.48 (0.43)	0.46 (0.35)	0.71 (0.5)	78.42 (1.88)
total distance	G2	0.07 (0.12)	6.18 (1.38)	8.29 (1.45)	0 (0)	10.74 (1.67)	0 (0)	74.71 (2.5)

Comment on additional file 3:

Model fitting revealed that the epiline was a significant factor to keep for explaining a portion of phenotypic variability for several traits. In adjusted tissue weights like heart, abdominal fat and testicle, this parameter was kept after the stepAIC in all generations. For the liver, it was only found significant in G2. For production traits, the epiline was found significant in precocity and in adjusted egg weight at every generation but not for the laying rate. Finally, in behavioural traits, the epiline factor was kept in the models for total distance and frequency at periphery in G1 and G2 only, whereas it was kept in G0 and G1 for duration at centre. Although the epiline appears as a significant factor in the aforementioned traits, the estimates of the portion of variability explained remain below 5% in all, and negligible in most (inferior to 1% in adjusted abdominal fat, adjusted liver weight, total distance, frequency at periphery and duration at centre).
Besides, for all the traits analysed, a substantial part of phenotypic variance remains uncaptured by our model, highlighting a strong limit in explanatory power and a limited confidence towards the estimates of the portion of phenotypic variability explained by the epiline. This is underlined by the large standard deviations found for all estimates, as well as

the limited correlation and determination coefficients. The interaction parameter that includes the epiline shows higher estimates according to the trait and generation, reaching up to 16.44% in adjusted egg weight in G2, or 18.41 in precocity in G0 for example, yet the model for these two traits fails to explain half of the overall phenotypic variability (respectively 41.21% and 38.22%).
