# Supplementary material for: Transmission of an environmental modification in quails across three generations: changes in the contribution to phenotypic variability
Source: Genet Sel Evol. 2026 Aug 10;58:57. doi: 10.1186/s12711-026-01079-0 (PMC13501780; doi:10.1186/s12711-026-01079-0)
Supplement: Supplementary file 4 [file 12711_2026_1079_MOESM4_ESM.pdf]

Additional file 4: Contribution of parameters to phenotypic variability per generation.

Figure S3 - Contribution of parameters (sex, family, epiline, reproductive status and their interactions) to phenotypic variability (in percentage) per generation. Interaction parameters were divided into two separate parameters: the interaction involving the epiline as one of the factors (interaction epi) and the interaction involving other parameters than the epiline (interaction non epi).

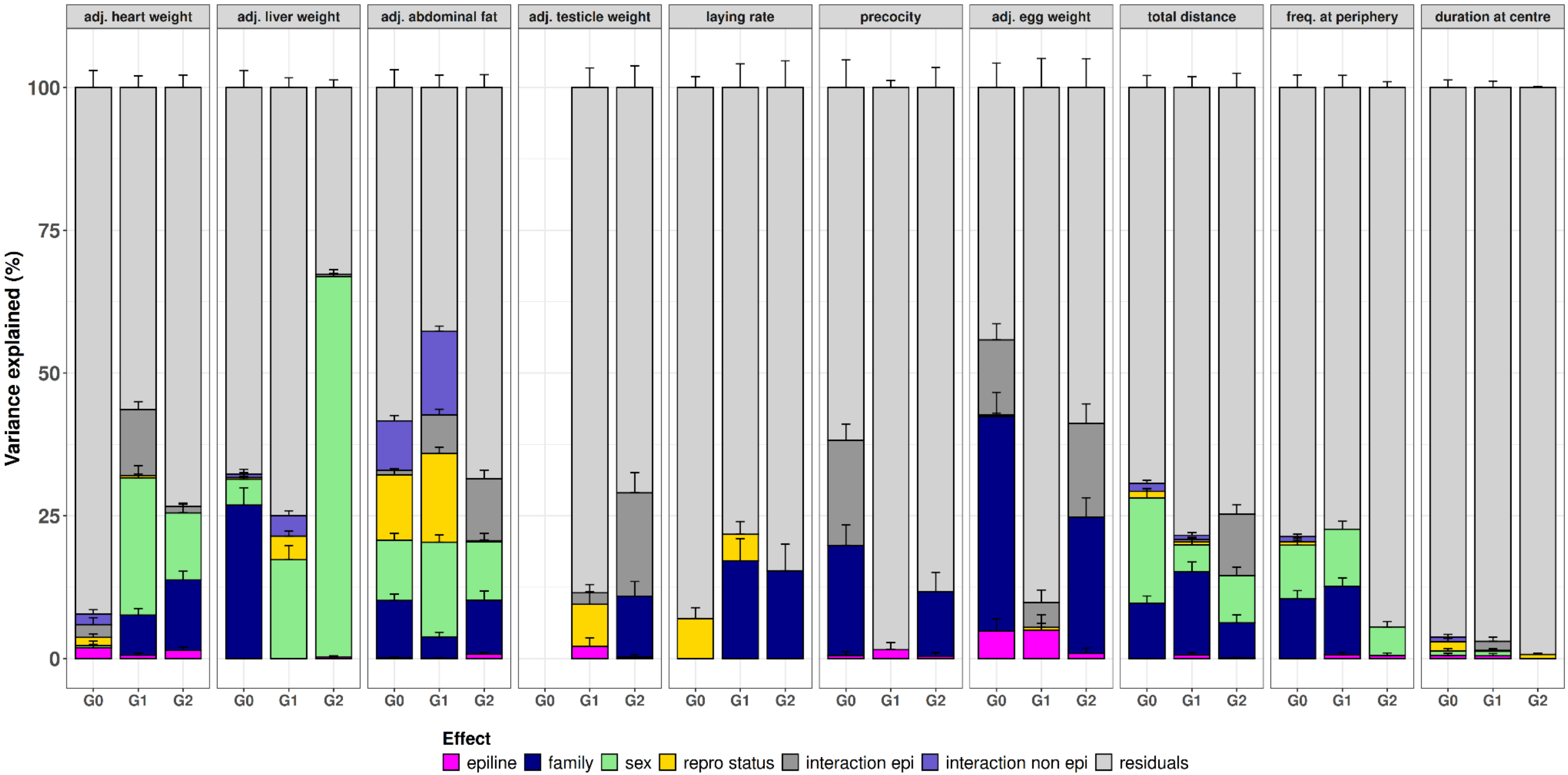
adj. heart weight
adj. liver weight
adj. abdominal fat
adj. testicle weight
laying rate
precocity
adj. egg weight
total distance
freq. at periphery
duration at centre
Variance explained (%)
100
75
50
25
0
G0
G1
G2
Effect
epiline
family
sex
repro status
interaction epi
interaction non epi
residuals

Figure S4 - Model robustness (R^2) and correlations after cross validations. The coefficient of determination R^2 (blue) and the correlation between observed and predicted values in the Test set (yellow) were determined in 100 cross validations, to evaluate the predictive performance of linear models. The results show the distribution of R^2 and correlation values in each of the 100 cross validations, and are displayed per generation (x-axis) for each phenotype.

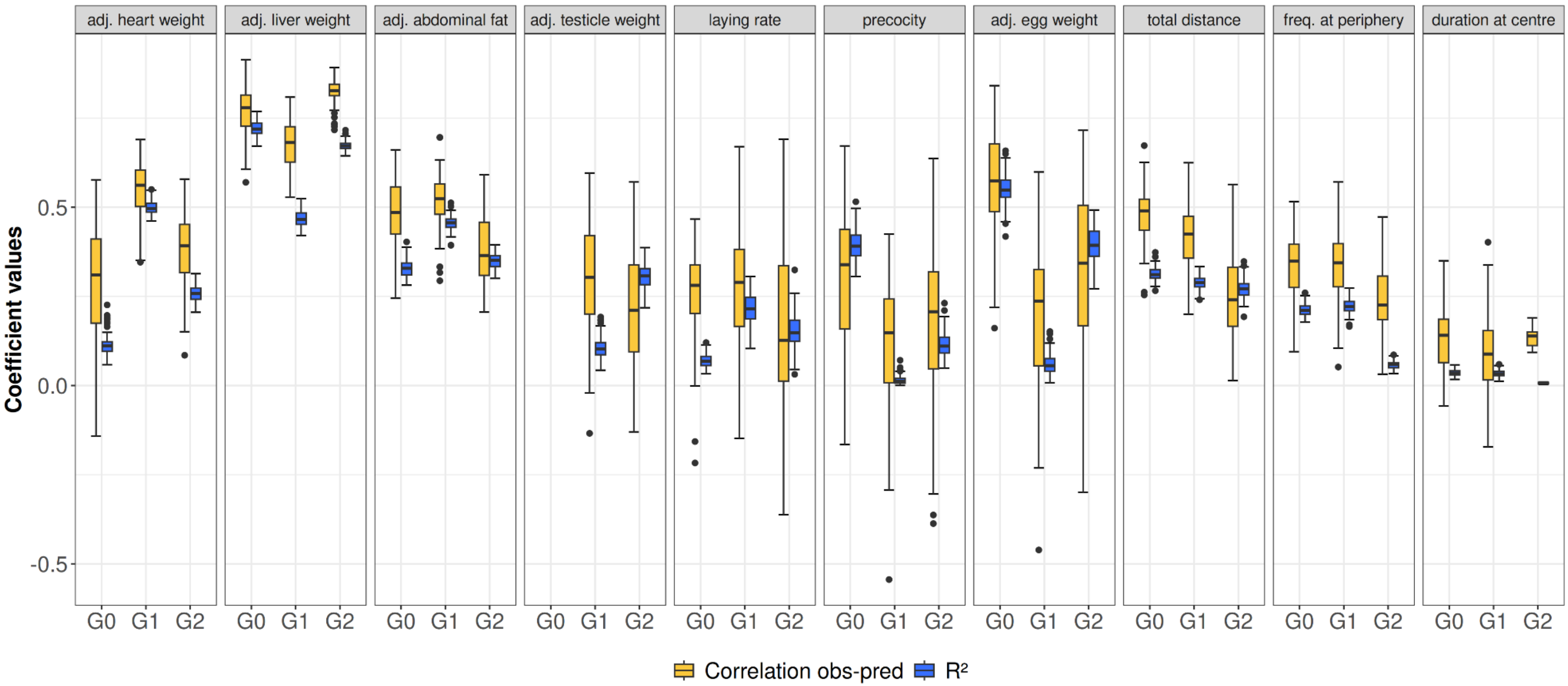

adj. heart weight
adj. liver weight
adj. abdominal fat
adj. testicle weight
laying rate
precocity
adj. egg weight
total distance
freq. at periphery
duration at centre
Coefficient values
0.5
0.0
-0.5
G0
G1
G2
Correlation obs-pred
R²
