# Supplementary material for: Transmission of an environmental modification in quails across three generations: changes in the contribution to phenotypic variability
Source: Genet Sel Evol. 2026 Aug 10;58:57. doi: 10.1186/s12711-026-01079-0 (PMC13501780; doi:10.1186/s12711-026-01079-0)
Supplement: Supplementary file 5 [file 12711_2026_1079_MOESM6_ESM.pdf]

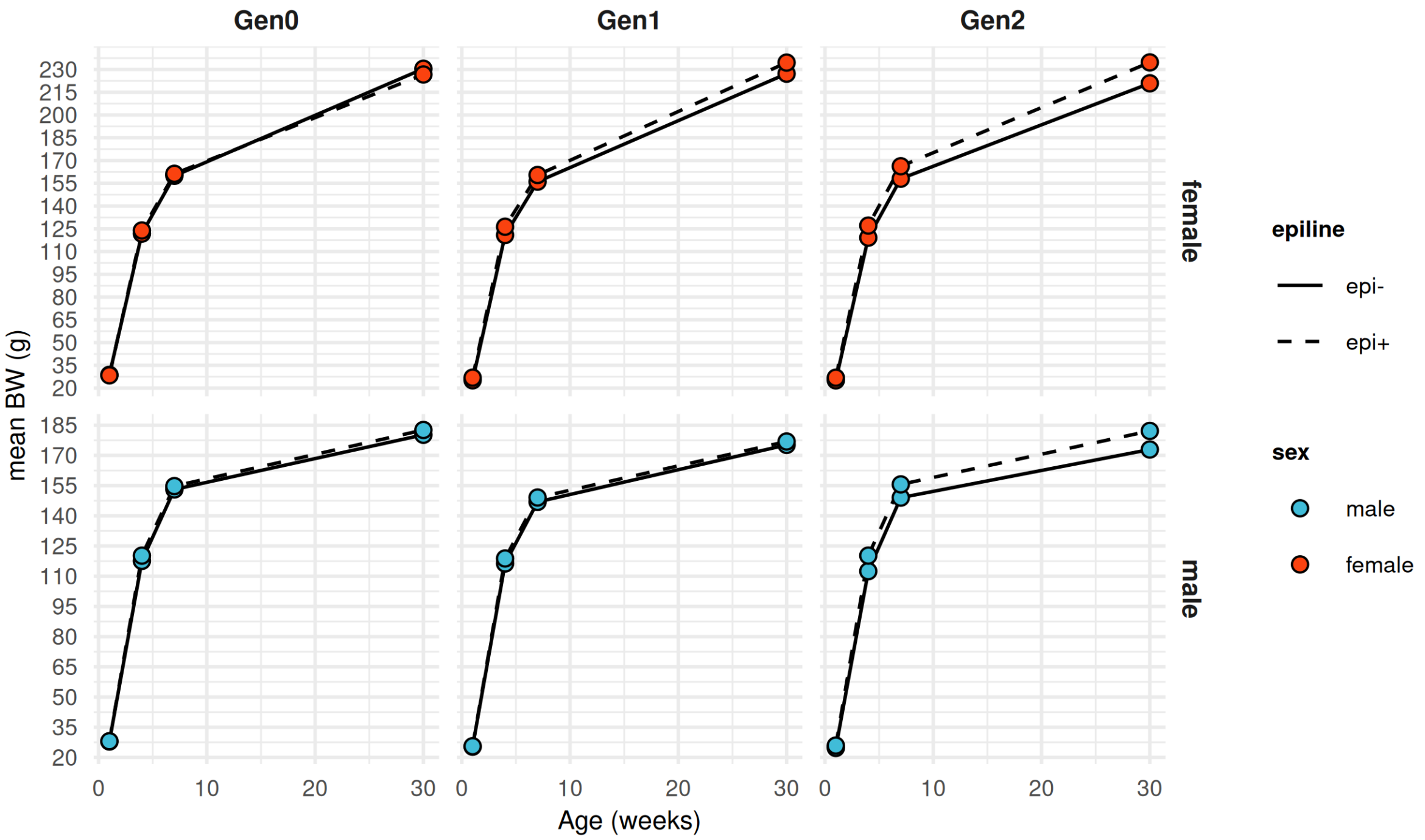

Gen0
Gen1
Gen2
mean BW (g)
230
215
200
185
170
155
140
125
110
95
80
65
50
35
20
185
170
155
140
125
110
95
80
65
50
35
20
0
10
20
30
Age (weeks)
female
male
epiline
epi-
epi+
sex
male
female
