# Supplementary material for: Transmission of an environmental modification in quails across three generations: changes in the contribution to phenotypic variability
Source: Genet Sel Evol. 2026 Aug 10;58:57. doi: 10.1186/s12711-026-01079-0 (PMC13501780; doi:10.1186/s12711-026-01079-0)

Additional file 7: Differences between epilines in adjusted tissue weights, production and behavioural traits.

Figure S6 - Adjusted tissue weights.
All tissue weights are adjusted by the adult body weight. The study was conducted for each combination of sex and generation independently. N values indicate sample sizes within each sex and epiline per generation. The asterisk indicates the level of significance of the Kruskal-Wallis test results after FDR correction for multiple testing, ns p-value > 0.05 (non significant),* p-value < 0.05, ** p-value < 0.01, *** p-value < 0.001 and **** p-value < 1e^{-4}.

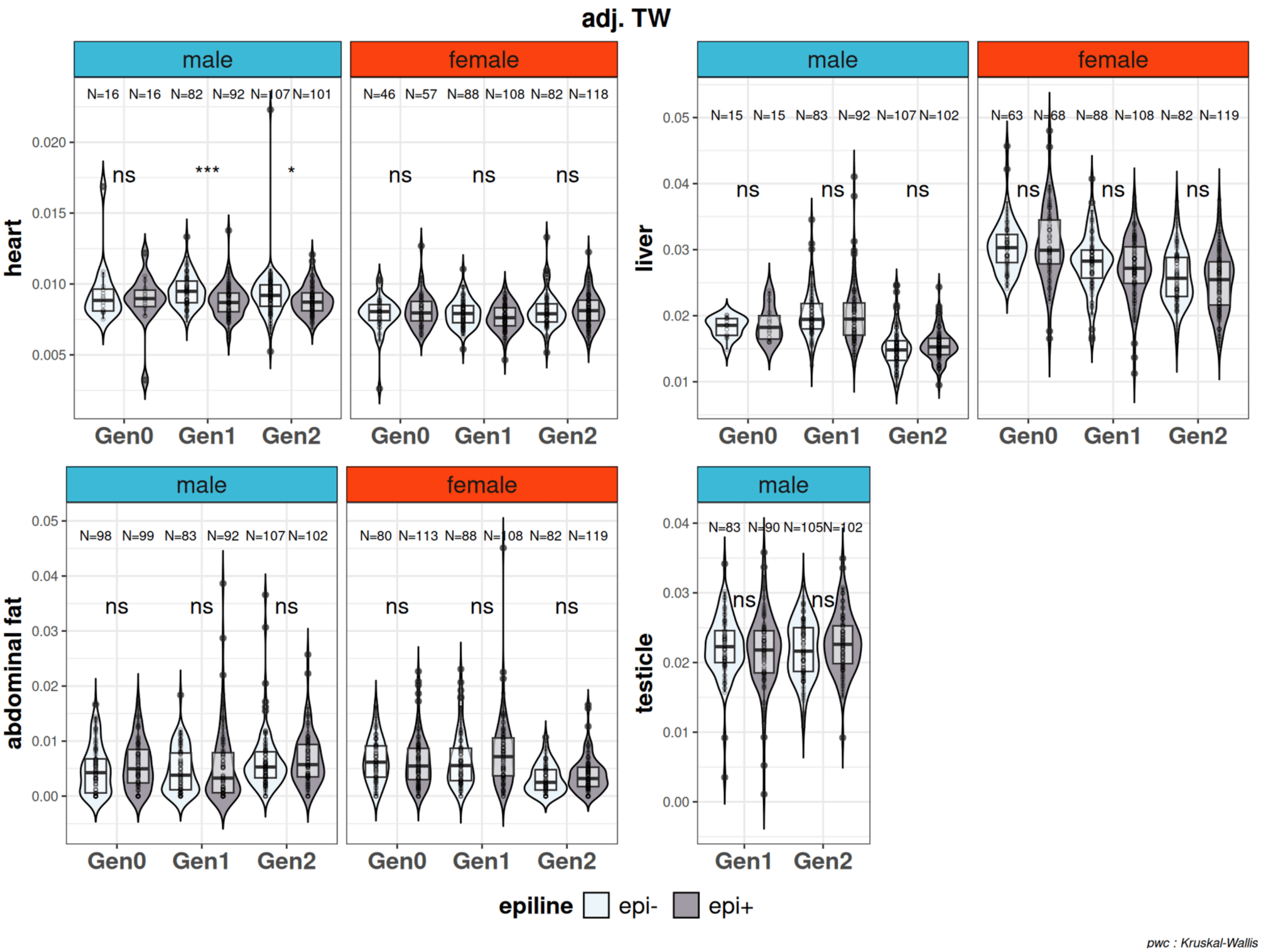

Figure S7 - Production traits.
Egg weight is adjusted by the adult body weight. The study was conducted for each generation independently. N values indicate sample sizes within each sex and epiline per generation. The asterisk indicates the level of significance of the Kruskal-Wallis test results after FDR correction for multiple testing, ns p-value > 0.05 (non significant), * p-value < 0.05, ** p-value < 0.01, *** p-value < 0.001 and **** p-value < $1e^{-4}$.

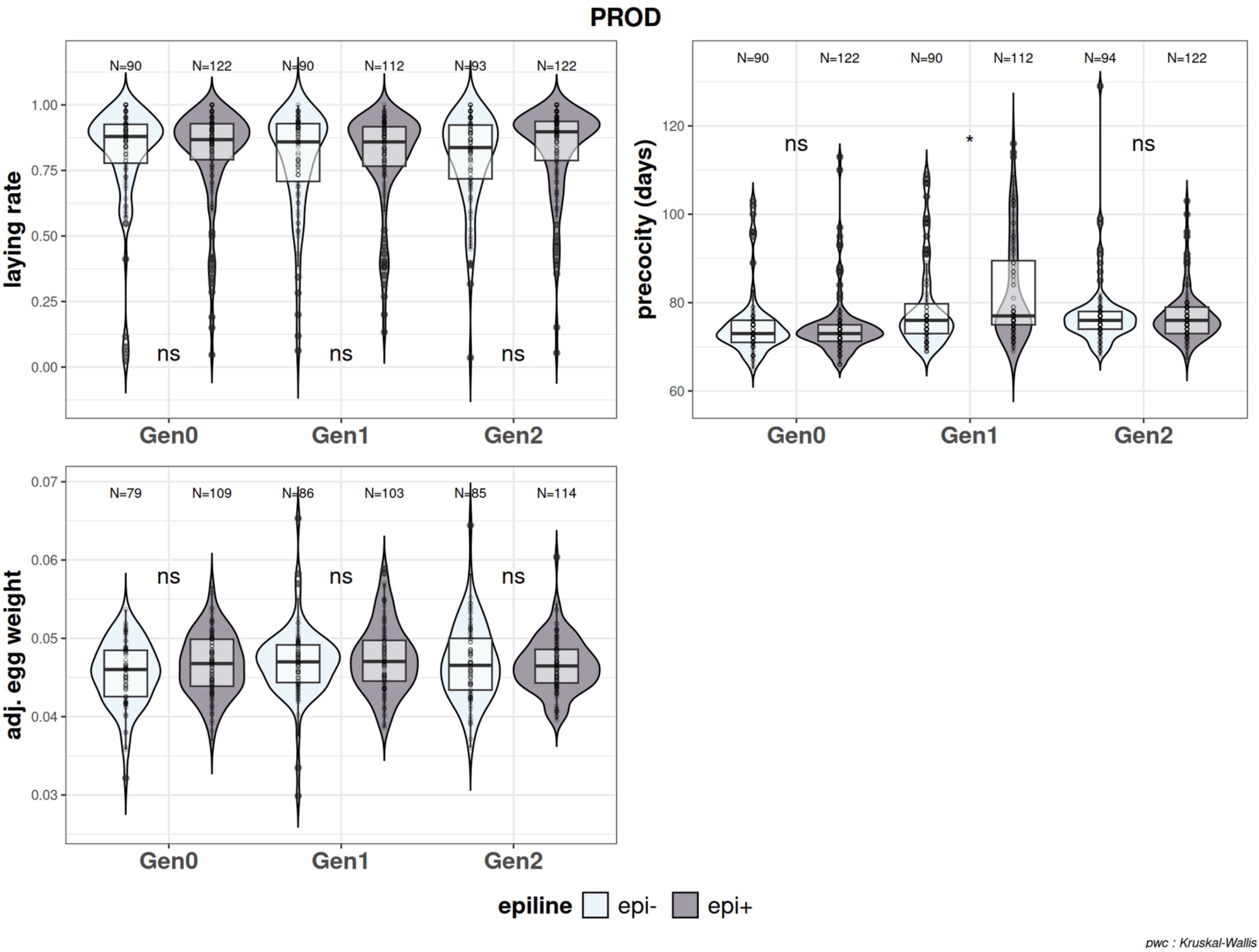

Figure S8 - Behavioural traits.
Total distance is expressed in millimetres. Duration at centre corresponds to the time spent in the inner area, expressed in seconds. The frequency at the periphery corresponds to the number of times the animal stepped out of the inner area. N values indicate sample sizes within each sex and epiline per generation. The asterisk indicates the level of significance of the Kruskal-Wallis test results after FDR correction for multiple testing, ns p-value > 0.05 (non significant), * p-value < 0.05, ** p-value < 0.01, *** p-value < 0.001 and **** p-value < $1e^{-4}$.

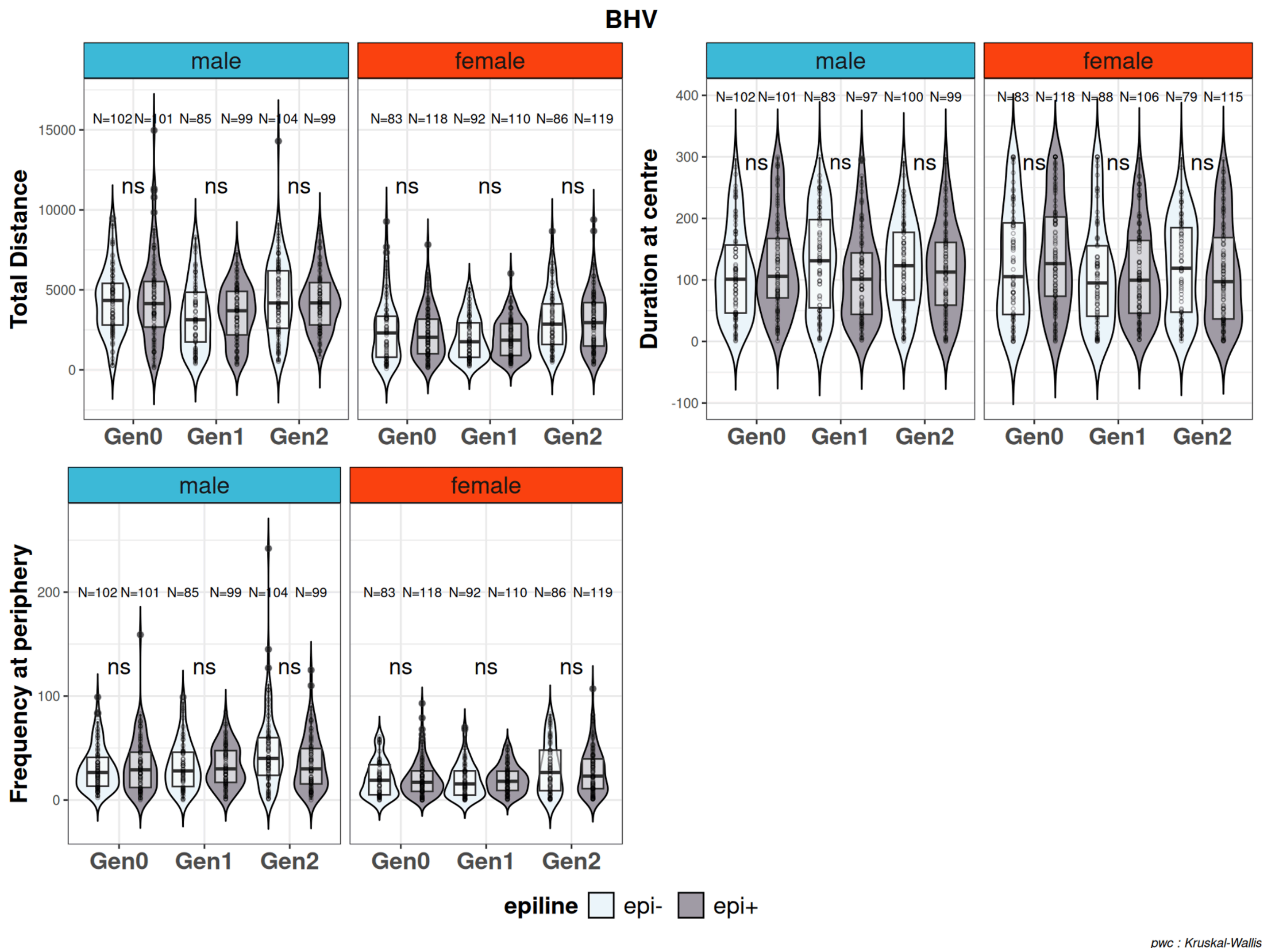

Supplement: Supplementary file 6 [file 12711_2026_1079_MOESM7_ESM.pdf]
